# xAtlas: scalable small variant calling across heterogeneous next-generation sequencing experiments

**DOI:** 10.1093/gigascience/giac125

**Published:** 2023-01-16

**Authors:** Jesse Farek, Daniel Hughes, William Salerno, Yiming Zhu, Aishwarya Pisupati, Adam Mansfield, Olga Krasheninina, Adam C English, Ginger Metcalf, Eric Boerwinkle, Donna M Muzny, Richard Gibbs, Ziad Khan, Fritz J Sedlazeck

**Affiliations:** Human Genome Sequencing Center, One Baylor Plaza, Baylor College of Medicine, Houston, TX 77030, USA; Human Genome Sequencing Center, One Baylor Plaza, Baylor College of Medicine, Houston, TX 77030, USA; Institute of Genomic Medicine, Columbia University, New York, NY 10027, USA; Human Genome Sequencing Center, One Baylor Plaza, Baylor College of Medicine, Houston, TX 77030, USA; Regeneron Pharmaceuticals, Inc., Tarrytown, NY 10591, USA; Human Genome Sequencing Center, One Baylor Plaza, Baylor College of Medicine, Houston, TX 77030, USA; Human Genome Sequencing Center, One Baylor Plaza, Baylor College of Medicine, Houston, TX 77030, USA; Human Genome Sequencing Center, One Baylor Plaza, Baylor College of Medicine, Houston, TX 77030, USA; Regeneron Pharmaceuticals, Inc., Tarrytown, NY 10591, USA; Human Genome Sequencing Center, One Baylor Plaza, Baylor College of Medicine, Houston, TX 77030, USA; Regeneron Pharmaceuticals, Inc., Tarrytown, NY 10591, USA; Human Genome Sequencing Center, One Baylor Plaza, Baylor College of Medicine, Houston, TX 77030, USA; Human Genome Sequencing Center, One Baylor Plaza, Baylor College of Medicine, Houston, TX 77030, USA; Human Genome Sequencing Center, One Baylor Plaza, Baylor College of Medicine, Houston, TX 77030, USA; Human Genetics Center, The University of Texas Health Science Center at Houston, Houston, TX 77030, USA; Human Genome Sequencing Center, One Baylor Plaza, Baylor College of Medicine, Houston, TX 77030, USA; Human Genome Sequencing Center, One Baylor Plaza, Baylor College of Medicine, Houston, TX 77030, USA; Human Genome Sequencing Center, One Baylor Plaza, Baylor College of Medicine, Houston, TX 77030, USA; Human Genome Sequencing Center, One Baylor Plaza, Baylor College of Medicine, Houston, TX 77030, USA

## Abstract

**Background:**

The growing volume and heterogeneity of next-generation sequencing (NGS) data complicate the further optimization of identifying DNA variation, especially considering that curated high-confidence variant call sets frequently used to validate these methods are generally developed from the analysis of comparatively small and homogeneous sample sets.

**Findings:**

We have developed xAtlas, a single-sample variant caller for single-nucleotide variants (SNVs) and small insertions and deletions (indels) in NGS data. xAtlas features rapid runtimes, support for CRAM and gVCF file formats, and retraining capabilities. xAtlas reports SNVs with 99.11% recall and 98.43% precision across a reference HG002 sample at 60× whole-genome coverage in less than 2 CPU hours. Applying xAtlas to 3,202 samples at 30× whole-genome coverage from the 1000 Genomes Project achieves an average runtime of 1.7 hours per sample and a clear separation of the individual populations in principal component analysis across called SNVs.

**Conclusions:**

xAtlas is a fast, lightweight, and accurate SNV and small indel calling method. Source code for xAtlas is available under a BSD 3-clause license at https://github.com/jfarek/xatlas.

## Findings

Over the past 20 years, multiple methods and approaches have surfaced, which aim to identify small variants across next-generation sequencing (NGS) short read data [1–3]. The improvement of methods to identify single-nucleotide variants (SNVs) and small insertions and deletions (indels) from NGS data remains an active area of research [1, 2]. Currently, small variant calling methods exceed sensitivity and precision of 99% for well-characterized samples [4]. There are ongoing efforts to refine small variant calls to address new demands in research and clinical domains, such as the need for reliably accurate and reproducible variant calls in clinical settings [5] and characterizing rare variants implicated in common diseases [6]. Considerable efforts have also been focused on increasing the computational efficiency of variant calling at scale. As with other secondary genomic analyses, recent small variant calling methods have leveraged more advanced computational techniques, including deep neural networks [7, 8] and specialized hardware [9]. Nevertheless, these advancements often come with demands for more compute or specialized hardware. Thus, faster and more lightweight variant calling methods are still desirable as they enable researchers with fewer available resources to identify variation across more samples or leverage variant calls as a quality control technique.

Runtime efficiency and speed are especially important when analyzing large datasets at the cohort or population level. The recent release of 3,202 samples across diverse populations at 30× whole-genome coverage by the 1000 Genomes Project (1KGP), for example, is an important step toward improving variant calling methods [10]. A per-sample SNV caller must scale to population-level variant detection by leveraging features such as genome VCF (gVCF) output and high per-sample variant call accuracy. Furthermore, it must cope with changes in sequencing technologies. As an example, The Trans-Omics for Precision Medicine program [11] has sequenced more than 93,000 whole genomes to date and aims to sequence at least 155,000 samples in total. The overall volume and heterogeneity of these data well exceed those benchmarks that are often used to establish specific methods. The NIST Genome In the Bottle Project (GIAB) has released multiple benchmarks and datasets [12] to provide an objective way to benchmark novel SNV callers [13] and other variant callers [14] on a single trio basis. These benchmarks are curated in part over a trio (HG002, HG003, HG004) and cover most of the human genome. These benchmarks enabled the improvement of SNV calling over the past years and the establishment of novel variant calling. Considerable efforts have also been focused on increasing the computational efficiency of sequence analysis and variant calling at scale, with GATK [15], DeepVariant [8], Illumina Dragen [9], and other state-of-the-art methods leveraging distributed software- and hardware-optimized technologies. However, these methods often require commitments to external infrastructure or internal technology development to be applied effectively, which may not be well suited for rapid turnaround times or cost-effective execution of variant calling at scale. The ideal variant caller should therefore allow fast performance and scalability on commodity computing hardware.

Here, we describe xAtlas, a lightweight and accurate single-sample SNV and small indel variant caller. xAtlas includes features that allow it to easily scale to population-scale sample sets, including support for CRAM [16] and gVCF file formats, minimal computational requirements, and fast runtimes. xAtlas demonstrates high accuracy when compared against GIAB reference benchmarks and favorable performance on large sample sets, such as those from 1KGP. xAltas is implemented as a command-line application written in C++ and is available under an open-source BSD license.

## Variant Calling Process

**Figure 1:**
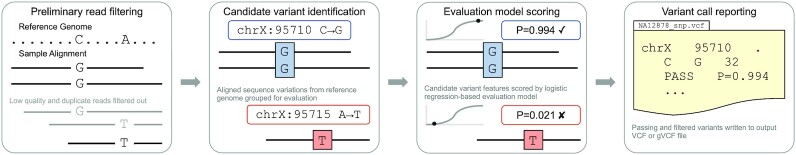
The major stages of xAtlas variant calling process.

Fig. 1 provides an overview of the main steps of xAtlas. xAtlas variant calling is performed in 3 stages: preliminary read filtering, collecting candidates for variant calling from aligned reads, and evaluating whether to call or report each candidate variant. First, reads that are marked as unmapped, are duplicate reads, or have a mapping quality below a user-configurable minimum threshold are filtered out from further processing. Next, candidate variants are identified by aggregating per-read sequence variations from the reference genome with matching left-aligned genomic coordinates.

While collecting candidate variants to evaluate, xAtlas also records values for a number of sequence and alignment features associated with each candidate variant, including mean base quality value, mean alignment quality for reads covering the candidate variant's genomic position, and coverage counts for reads supporting the candidate variant allele. These values are provided either to a SNV or to an indel logistic regression model to assign a score of the candidate variant's likelihood of being a real variant. This likelihood score, along with separate cutoffs for low alignment quality and other filtering criteria, determines whether the variant is called and whether a called variant is filtered. A variant's likelihood score also provides a proxy measure of confidence of the variant's called genotype, as xAtlas does not calculate phred-scaled genotype quality (GQ) scores directly.

xAtlas allows the user to reconfigure the logistic regression intercepts and variable coefficients of these models. After assigning variant confidence scores from the logistic regression models and applying VCF filters, xAtlas genotypes and reports the candidate variant in the output VCF. SNVs and indels are written to separate output VCF files.

xAtlas' variant evaluation model excludes some techniques that are employed by other single-sample variant callers. Notably, xAtlas does not perform local realignment around candidate variant regions, which contributes in part to reduced application runtimes. xAtlas also attempts to call no more than 1 SNV or indel variant allele at a given genomic locus. Despite these model limitations, xAtlas nonetheless achieves a high degree of variant sensitivity and precision.

xAtlas is implemented as a command-line application written in C++ that employs HTSlib [17] to handle HTS alignment and variant file formats. Input sample alignment files may be in either BAM [18] or CRAM [16] format, and output variant calls are written in VCF format [19]. xAtlas includes options for multithreading and writing output in gVCF format, in which xAtlas includes additional VCF entries to capture coverage information for regions between called variants.

## Variant Call Assessment with NIST Genome in a Bottle HG002 Benchmark Dataset

xAtlas variant calling accuracy was assessed by measuring the concordance between xAtlas variant calls on reference alignments and corresponding benchmark call sets from release 4.2.1 of the NIST GIAB [13]. Table 1 summarizes the results of xAtlas variant calling on the GIAB Ashkenazi trio reference samples. xAtlas variant calls on an HG002 sample at 60× mean whole-genome coverage had a precision of 98.43% and a sensitivity of 99.11% for SNVs and a precision of 94.85% and a sensitivity of 86.28% for indels, relative to SNVs and indels in the GIAB HG002 benchmark call set within high-confidence regions. xAtlas achieved these results in 1.9 hours.

**Table 1:** Precision and sensitivity of xAtlas SNV and indel calls on Ashkenazi trio reference samples from NIST GIAB relative to GIAB v4.2.1 benchmark small variants

Sample	Coverage	SNV precision	SNV sensitivity	Indel precision	Indel sensitivity
HG002	60.72	0.9843	0.9911	0.9485	0.8628
HG003	53.09	0.9855	0.9909	0.9489	0.8603
HG004	61.47	0.9854	0.9911	0.9491	0.8645

## xAtlas Performance Comparison across NA12878 Benchmark Datasets

**Figure 2:**
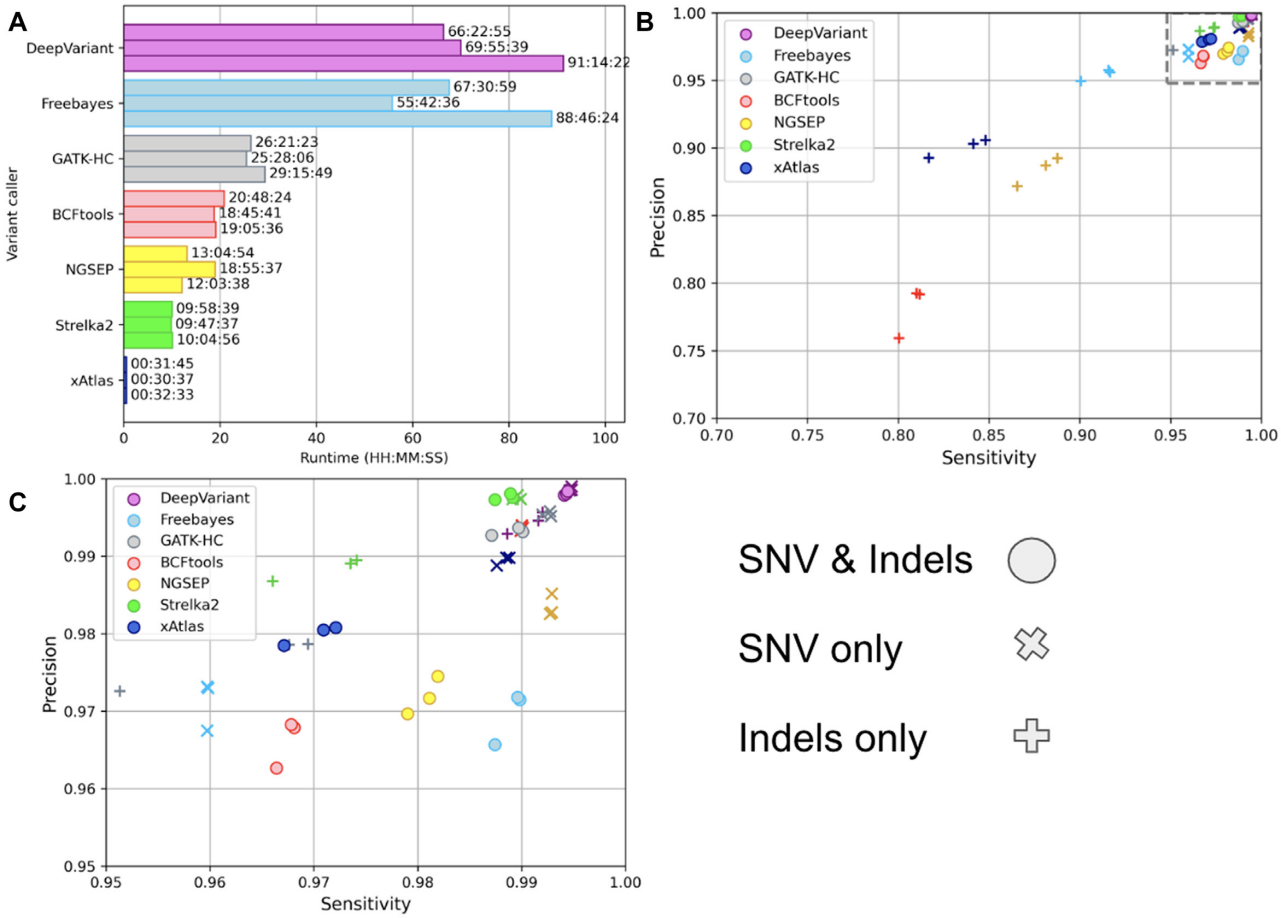
(A) Variant caller runtimes of xAtlas and 4 other small variant callers (single-threaded operation) on 3 Illumina HiSeq X NA12878 whole-genome samples at 30× average coverage. (B, C) Precision vs. sensitivity of passing variants called for these samples concordant with GIAB v4.2.1 NA12878 high-confidence variants. Marker shape indicates values per variant caller for SNVs only (“X”), indels only (“+”), or SNVs and indels together (filled circles).

Next, we assessed xAtlas performance in terms of speed and accuracy across another GIAB benchmark sample, NA12878 [20]. xAtlas, Freebayes [21], GATK HaplotypeCaller [15], DeepVariant [22], NGSEP SingleSampleVariantsDetector [23], Strelka2 [24], and a Samtools- and BCFtools-based [18] variant calling pipeline were benchmarked on 3 whole-genome NA12878 samples sequenced on the Illumina HiSeqX platform at 30× average whole-genome coverage. xAtlas demonstrated the lowest runtimes of between 0.510 and 0.543 hours across the 3 NA12878 samples (Fig. 2A) with concordance F-measures of between 0.9727 and 0.9764 when compared to GIAB v4.2.1 benchmark small variants in high-confidence regions (Fig. 2B).

## Variant Call Set Generation on 3,202 Samples from the 1000 Genomes Project

To assess the performance of xAtlas on a large-scale NGS dataset, xAtlas was run on a dataset of 3,202 samples from the 1KGP [10], with a mean whole-genome coverage of 34.17 across the sample set. For the 1KGP dataset, xAtlas was run in single-threaded operation for each sample, in parallel on an HPC cluster, with an average runtime of 1.7 hours per sample and a cumulative runtime of 5,437.4 hours for the 3,202 samples. An average of 4.585 M passing SNVs and 880.3 K passing indels were called per sample, for a total of 17.5 B passing variants called across all samples.

**Figure 3:**
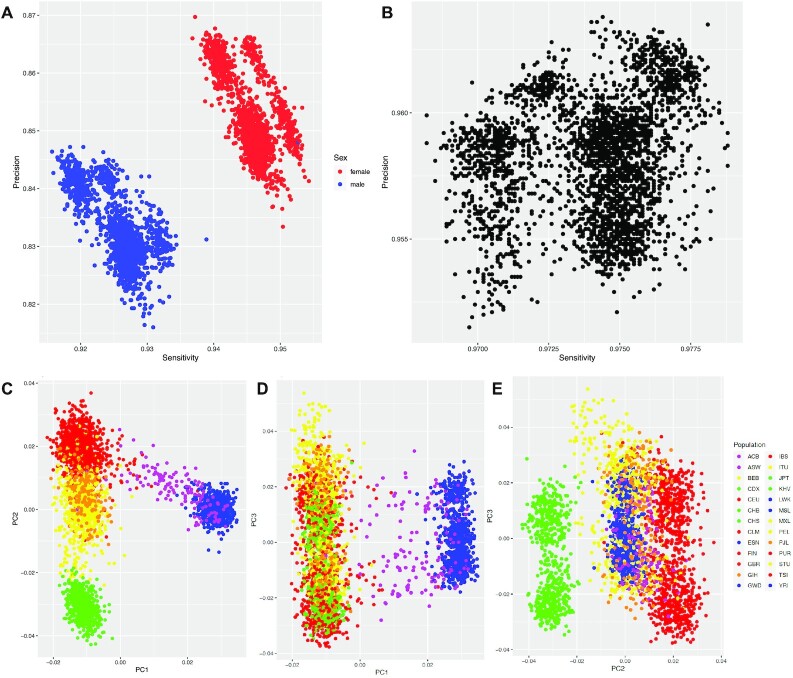
1000 Genome comparison: Sensitivity and precision of xAtlas variant calls compared against SNVs and indels from a high-coverage Illumina phased panel generated on 3,202 samples from the 1000 Genomes Project, using (A) all passing variants on chromosomes 1–22 and X and (B) passing variants within GIAB HG002 high-confidence variant regions on chromosomes 1–22. PCA plots of chromosome 22 SNVs with MAF ≥ 0.05 called by xAtlas on these 3,202 samples, showing (C) PC1 vs. PC2, (D) PC1 vs. PC3, and (E) PC2 vs. PC3.

First we assessed the concordance between xAtlas and published SNP calls that were curated over the entire 3,202 samples [10]. For this, we utilized rtgtools (see Methods) and plotted the concordance of xAtlas to the publicly available call set. Fig. 3A shows the concordance (mean: precision 84.26 and sensitivity 93.57) for each sample across the entire genome. Interestingly, we identified a gender bias in the samples from xAtlas based on the ChrX and Y variant calling. Fig. 3B restricts the comparison to the GIAB tier1 SNV regions (version 4.2.1) that shows an unbiased result with very high concordance from xAtlas (mean: precision 95.82 and sensitivity 97.41), thus showing that xAtlas within only 5,437 hours of compute could achieve highly accurate results across these 3,202 samples.

The transition vs. transversion (Ti/Tv) ratios of passing SNVs called in autosomal chromosomes for the 1KGP dataset had a mean of 1.928 with a standard deviation of 0.0094. Within the Ashkenazi trio, when limited to GIAB high-confidence variant calling regions determined for the trio, the average Ti/Tv ratio for passing variants in the Ashkenazi trio was 2.08. This is in agreement with the expectation of a Ti/Tv ratio of 2.07 to 2.10 for whole-genome sequencing in humans [25]. GIAB high-confidence regions for the Ashkenazi trio cover an average of 83.6% of nonmasked autosomal sequences in the GRCh38 reference genome.

Principal component analysis (PCA) of SNVs called across these samples supports variant call accuracy across diverse populations. Fig. 3 shows a PCA plot of the first 2 principal components of samples from the 3,202-sample dataset for SNVs called on chromosome 22 with a minor allele frequency (MAF) of at least 0.05. Samples within the same or related populations are found in identifiable clusters, while population clusters show proximity to related populations (Fig. 3C). Population-level clustering is also observed when plotting principal components 1 vs. 3 (Fig. 3D), as well as principal components 2 vs. 3 (Fig. 3E).

To summarize, xAtlas has demonstrated a combination of computational efficiency and variant call accuracy. Sensitivity and precision rates for both SNVs and indels called by xAtlas rank among those of other variant calling methods that have been used in practice. xAtlas has permitted fast and cost-effective variant analysis across multiple projects at the BCM-HGSC consisting of tens of thousands of whole-genome samples. For small- or large-scale variant analysis, xAtlas can be scaled and run in compute environments ranging from a single laptop to large HPC clusters or arrays of cloud instances. With the ability to generate VCFs and gVCF-formatted variant call sets in terms of minutes or hours per sample, development of new variant analysis methods can also be carried out with rapid turnaround rates.

## Methods

### Variant call quality assessment

xAtlas is a command-line C++ application that employs HTSlib [18] to handle alignment and variant call file formats. Input sample alignment files may be in either BAM [18] or CRAM [16] format. When writing output in VCF format [19], xAtlas may optionally include nonvariant VCF entries spanning regions not covered by variants formatted in gVCF format [26] to facilitate downstream multisample variant analyses. The application also may be built with multithreading support, which allows the processes of reading the input alignment file, processing SNVs, and processing indels to be handled each in a separate thread.

### Variant detection and evaluation

xAtlas variant calling is performed in the following high-level stages: preliminary read filtering, collecting candidates for variant calls from the alignment file, evaluating each candidate variant, and reporting candidate variants (Fig. 1).

Preliminary read filtering is performed to filter out uninformative reads. As reads are scanned from the input alignment, reads marked as unmapped, as duplicate reads, or having a mapping quality score below a minimum threshold, with a default of 1, are filtered out from further evaluation.

Candidate sequence variations are then collected from the unfiltered reads and grouped for variant calls. To aggregate candidates, sequence variations are identified within each read by locating the coordinates at which sequences differ from the provided reference genome. The SAM format's CIGAR string, which defines the edit operations between the read's sequence and the reference sequence at its mapped position, is used to determine variant coordinates. SNVs are defined as point differences between reference and aligned sample sequences within the spans of CIGAR match operators. Variant alleles are assigned reference coordinates that correspond to its parsimonious representation within the alignment, as defined by Tan et al. [27].

While collecting candidate variants to evaluate, xAtlas also records a number of values associated with sequence and alignment features, such as average base quality score across supporting reads, for each candidate variant. These values are then fed to 1 of 2 logistic regression models, for either SNVs or indels, to calculate the probability that the candidate variant is a real variant based on the assessed features. Table 2 shows the features assessed for each candidate variant and the default model parameters assigned to these features. Thresholds for both confidence scores derived from logistic regression probabilities and specific features determine whether a candidate variant will be called and if a called variant will be called as a filtered variant in the output VCF file. Other filters may also be applied to candidate variants based on other features not evaluated by the candidate evaluation models, such as if there are too few reads supporting the candidate variant allele. xAtlas allows the user to redefine the logistic regression intercepts and variable coefficients of these models with values that may be derived from retraining on new samples.

**Table 2:** Default values set by xAtlas for the SNV (a) and indel (b) logistic regression model parameters for scoring candidate variants

(a) SNV logistic regression model		
Parameter	Default coefficient	Description
intercept	−6.6404	Logistic regression intercept
ratio_score	11.1192	Score of ratio of reads supporting the candidate SNV vs. all reads covering the SNV
base_qual	0.25579	Average base quality at candidate SNV position across supporting reads
mean_avnqs	0.12896	Average base quality within 5 bases of candidate SNV position across supporting reads
rel_pos	0.69106	Relative position of the candidate SNV within each read averaged across all supporting reads
titv	0.48511	SNV is transition (1) or transversion (0)
**(b) Indel logistic regression model**		
intercept	−7.1085	Logistic regression intercept
ratio_score	6.22804	Score of ratio of reads supporting the candidate indel vs. all reads covering the indel
strand_dir	2.21407	Indel is supported by reads on both positive and negative strands (true: 1; false: 0)
mean_avnqs	0.07777	Average base quality within 5 bases of candidate indel position across supporting reads
seq_entropy	0.1479	Insertion or deletion sequence entropy calculation
mean_var_rate	−2.13305	Count of variant sequences in each read averaged across all supporting reads

After assigning confidence scores to candidate variants and applying filters, xAtlas then determines the most likely genotype and reports the candidate variant in the VCF. A variant call is reported in the VCF only if the candidate's logistic regression value is greater than an adjustable cutoff, with a default value of 0.25. If multiple variants may be reported at the same position, xAtlas reports only the variant at that position with the greatest number of reads supporting the variant sequence. For SNVs, if there are still multiple candidates tied for the greatest number of supporting reads, the candidate variant with the highest logistic regression value is then selected. xAtlas assigns the genotype 1/1, 0/1, or 0/0 to called variants. For indels, genotypes are assigned based on cutoffs for the ratio of reads supporting the variant allele to the total number of reads overlapping the indel. For SNVs, each SNV is assigned the genotype with the highest genotype likelihood as determined by xAtlas.

### Retraining candidate variant evaluation models

The logistic regression model retraining performed as part of this study was performed by building sets of positive and negative examples of variant sites from pairs of sample alignments and using subsets of these variant sites in logistic regression model fitting. The set of all possible candidate variant sites and the values that xAtlas supplies to the SNV and indel logistic regression models were compiled for each sample. Subsets of positive and negative variant site examples were then derived from this set based on variant site overlaps with a truth set of high-confidence variants and with high-confidence variant regions. Positive variant sites were selected from variant sites present in both technical replicates, overlapping the NIST high-confidence variants, and restricted to the NIST high-confidence regions. Two types of negative variant sample sites were compiled, where variant sites are either present in both technical replicates or present in only 1 of the 2 replicates, with both types restricted to the NIST high-confidence regions but not overlapping the NIST high-confidence variants. Each of these comprised half of the negative example variant sites in assembled training and testing sets. Training and testing sets were compiled as nonoverlapping sets of 10,000 randomly sampled positive and negative variant site examples, with a 1:1 ratio of positive vs. negative examples in each set. Logistic regression model fitting using these training and testing sets was performed using the LogisticRegression classifier from scikit-learn [28].

## xAtlas Performance Assessment on GIAB and 1000 Genomes Project

All GIAB and 1000 Genomes Project sample alignments used as input for xAtlas were in CRAM format and aligned to the GRCh38 human reference genome. xAtlas runs were performed on a Linux HPC cluster at the BCM-HGSC, with runtimes measured using system time utilities on the HPC cluster. xAtlas command-line invocations included the “–gvcf” and “–bgzf” output options.

### GIAB benchmark comparisons

xAtlas variant call precision and sensitivity measurements were calculated by the vcfeval function in RTG-tools [29] version 3.9.1. SNVs for HG002, HG003, and HG004 derived from NIST Genome in a Bottle release 4.2.1 were used as truth sets in vcfeval comparisons, with corresponding GIAB high-confidence variant region BED files used as evaluation regions in these comparisons.

### 1000 Genomes project data analysis

Principal component analysis was carried out by constructing a project-level VCF (pVCF) file of SNVs called by xAtlas on 3,202 samples from the 1000 Genomes Project (1000GP) sample set and estimating principal components based on the called genotype of passing variants across the sample set at each genetic locus. Principal component analysis on the final pVCF was performed by PLINK (RRID:SCR_001757) [30] version 1.9.

We compared the publicly available 1000 Genomes catalog to xAtlas using RTG-tools [29] version 3.9.1 one time across the entire genome and one time with GIAB BED files (release 4.2.1).

The project-level VCF for the 3,202 1000GP samples was constructed in a few phases. First, the union of all genetic loci to be considered for principal component estimation was determined. This was defined to be all genetic loci for which xAtlas called a passing SNV on at least 1 sample with the 3,202-sample set and for which the MAF of the called variant within the 3,202-sample set was at least 0.05. Next, a pVCF file was constructed by combining the xAtlas-called genotypes for the 3,202 samples into a single file in VCF format. Each VCF record in the pVCF file represents a genetic locus from the previously determined set of all xAtlas-called genetic loci across the sample set. Each of the 3,202 samples is represented in the pVCF by a sample-level column containing xAtlas-called genotypes from the sample-level VCF across all genetic loci recorded in the pVCF. While this pVCF was constructed using purpose-built scripts, other utilities that can also be used to construct pVCF files include GLnexus [31], which includes support for gVCFs created by xAtlas.

## Availability of Supporting Source Code and Requirements

xAtlas source code and instructions may be downloaded from https://github.com/jfarek/xatlas. HTSlib (https://github.com/samtools/htslib) is required for building xAtlas.

- Project name: xAtlas
- Project homepage: https://github.com/jfarek/xatlas
- Operating system(s): Platform independent
- Programming language: C++
- Other requirements: HTSlib 1.3 or higher
- License: BSD 3-Clause
- RRID: SCR_022987

## Data Availability

NIST GIAB data were obtained from [32]. 1000 Genomes Project data were obtained from [33]. 1000 Genomes Project SNV data from high coverage [34]. All supporting data and materials are available in the *GigaScience* GigaDB database [35].

## Abbreviations

1KGP: 1000 Genomes Project; GIAB: Genome In the Bottle Project; GQ: genotype quality; gVCF: genome VCF; MAF: minor allele frequency; NGS: next-generation sequencing; PCA: principal component analysis; pVCF: project-level VCF; SNV: single-nucleotide variant; Ti/Tv: transition vs. transversion.

## Funding

This work has been supported by NHGRI Centers for Common Disease Genomics grant 5UM1HG008898-02.

## Conflicts of Interest

The authors declare no conflicts of interest.

## Authors' Contributions

Application development and code implementation: J.F. and D.H. Sample analysis: J.F., Y.Z., A.P., A.M., O.K., and A.E. Project coordination: W.S., R.G., Z.K., and F.S. All authors contributed to the manuscript writing.

## Supplementary Material

giac125_GIGA-D-21-00249_Original_Submission

giac125_GIGA-D-21-00249_Revision_1

giac125_GIGA-D-21-00249_Revision_2

giac125_GIGA-D-21-00249_Revision_3

giac125_GIGA-D-21-00249_Revision_4

giac125_Response_to_Reviewer_Comments_Original_Submission

giac125_Response_to_Reviewer_Comments_Revision_1

giac125_Response_to_Reviewer_Comments_Revision_2

giac125_Response_to_Reviewer_Comments_Revision_3

giac125_Reviewer_1_Report_Original_Submission Ruibang Luo -- 9/19/2021 Reviewed

giac125_Reviewer_2_Report_Original_Submission Jorge Duitama -- 10/4/2021 Reviewed

giac125_Reviewer_2_Report_Revision_1 Jorge Duitama -- 3/7/2022 Reviewed

giac125_Reviewer_4_Report_Revision_1 Lachlan Coin -- 8/28/2022 Reviewed
