# Supplementary material for: xAtlas: scalable small variant calling across heterogeneous next-generation sequencing experiments
Source: Gigascience. 2023 Jan 16;12:giac125. doi: 10.1093/gigascience/giac125 (PMC9841152; doi:10.1093/gigascience/giac125)
Supplement: giac125_GIGA-D-21-00249_Original_Submission [file giac125_giga-d-21-00249_original_submission.pdf]

GigaScience

xAtlas: Scalable small variant calling across heterogeneous next-generation sequencing experiments

--Manuscript Draft--

**Manuscript Number:**	GIGA-D-21-00249
**Full Title:**	xAtlas: Scalable small variant calling across heterogeneous next-generation sequencing experiments
**Article Type:**	Technical Note
**Funding Information:**	National Institutes of Health (5UM1HG008898-02) — Dr Richard Gibbs
**Abstract:**	Background: The growing volume and heterogeneity of next-generation sequencing (NGS) data complicate the further optimization of identifying DNA variation, especially considering that curated high-confidence variant call sets frequently used to validate these methods are generally developed from the analysis of comparatively small and homogeneous sample sets.<br><br>Findings: We have developed xAtlas, a single-sample variant caller for single nucleotide variants (SNV) and small insertions and deletions (indels) in NGS data. xAtlas features rapid runtimes, support for CRAM and gVCF file formats, and retraining capabilities. xAtlas reports SNVs with 99.75% recall and 98.88% precision across a reference HG002 sample at 60x whole-genome coverage in less than 2 CPU hours. Applying xAtlas to 3,202 samples at 30x whole-genome coverage from the 1000 Genomes Project achieves an average runtime of 1.7 hours per sample and a clear separation of the individual populations in principal component analysis across called SNVs.<br><br>Conclusions: xAtlas is a fast, lightweight, and accurate SNV and small indel calling method. Source code for xAtlas is available under a BSD 3-clause license at https://github.com/jfarek/xatlas
**Corresponding Author:**	Fritz Sedlazeck<br>Baylor College of Medicine<br>UNITED STATES
**Corresponding Author Secondary Information:**	
**Corresponding Author's Institution:**	Baylor College of Medicine
**Corresponding Author's Secondary Institution:**	
**First Author:**	Jesse Farek
**First Author Secondary Information:**	
**Order of Authors:**	Jesse Farek<br>Daniel Hughes<br>William Salerno<br>Yiming Zhu<br>Aishwarya Pisupati<br>Adam Mansfield<br>Olga Krasheninina<br>Ginger Metcalf<br>Eric Boerwinkle<br>Donna Muzny

Powered by Editorial Manager® and ProduXion Manager® from Aries Systems Corporation

	Richard Gibbs
	Ziad Khan
	Fritz Sedlazeck
**Order of Authors Secondary Information:**	
**Additional Information:**	
**Question**	**Response**
Are you submitting this manuscript to a special series or article collection?	No
**Experimental design and statistics**<br><br>Full details of the experimental design and statistical methods used should be given in the Methods section, as detailed in our Minimum Standards Reporting Checklist. Information essential to interpreting the data presented should be made available in the figure legends.<br><br>Have you included all the information requested in your manuscript?	Yes
**Resources**<br><br>A description of all resources used, including antibodies, cell lines, animals and software tools, with enough information to allow them to be uniquely identified, should be included in the Methods section. Authors are strongly encouraged to cite Research Resource Identifiers (RRIDs) for antibodies, model organisms and tools, where possible.<br><br>Have you included the information requested as detailed in our Minimum Standards Reporting Checklist?	Yes
**Availability of data and materials**<br><br>All datasets and code on which the conclusions of the paper rely must be either included in your submission or deposited in publicly available repositories (where available and ethically	Yes

Powered by Editorial Manager® and ProduXion Manager® from Aries Systems Corporation

appropriate), referencing such data using a unique identifier in the references and in the "Availability of Data and Materials" section of your manuscript.<br><br>Have you have met the above requirement as detailed in our Minimum Standards Reporting Checklist?	

Manuscript Click here to access/download;Manuscript;manuscript.docx

xAtlas: Scalable small variant calling across heterogeneous next-generation sequencing experiments

Jesse Farek[1,*], Daniel Hughes[1,2,*], William Salerno[3,1], Yiming Zhu[1], Aishwarya Pisupati[1], Adam Mansfield[3,1], Olga Krasheninina[3,1], Ginger Metcalf[1], Eric Boerwinkle[1,4], Donna M Muzny[1], Richard Gibbs[1], Ziad Khan[1], Fritz J. Sedlazeck[1]

[1] Baylor College of Medicine Human Genome Sequencing Center, Houston, Texas, 77030
[2] Columbia University, New York, New York, 10027
[3] Regeneron Pharmaceuticals, Inc., Tarrytown, NY, 10591
[4] Human Genetics Center, The University of Texas Health Science Center at Houston, Houston, Texas, 77030,USA.

*: equal contribution
Corresponding: Jesse.Farek@bcm.edu, fritz.sedlazeck@bcm.edu

Abstract

Background: The growing volume and heterogeneity of next-generation sequencing (NGS) data complicate the further optimization of identifying DNA variation, especially considering that curated high-confidence variant call sets frequently used to validate these methods are generally developed from the analysis of comparatively small and homogeneous sample sets.

Findings: We have developed xAtlas, a single-sample variant caller for single nucleotide variants (SNV) and small insertions and deletions (indels) in NGS data. xAtlas features rapid runtimes, support for CRAM and gVCF file formats, and retraining capabilities. xAtlas reports SNVs with 99.75% recall and 98.88% precision across a reference HG002 sample at 60x whole-genome coverage in less than 2 CPU hours. Applying xAtlas to 3,202 samples at 30x whole-genome coverage from the 1000 Genomes Project achieves an average runtime of 1.7 hours per sample and a clear separation of the individual populations in principal component analysis across called SNVs.

Conclusions: xAtlas is a fast, lightweight, and accurate SNV and small indel calling method. Source code for xAtlas is available under a BSD 3-clause license at https://github.com/jfarek/xatlas

Findings

Over the past 20 years multiple methods and approaches have surfaced, which aim to identify small variants across next-generation sequencing (NGS) short read data [1–3]. The improvement of methods to identify single nucleotide variants (SNVs) and small insertions and deletions (indels) from NGS data remains an active area of research [1,2]. Despite multiple

best-practices small variant calling methods exhibiting sensitivity and precision exceeding 99% for well-characterized samples[4]. There are ongoing efforts to refine small variant calls to address new demands in research and clinical domains, such as the need for reliably accurate and reproducible variant calls in clinical settings [5] and characterizing rare variants implicated in common diseases [6]. Considerable efforts have also been focused on increasing the computational efficiency of variant calling at scale. As with other secondary genomic analyses, recent small variant calling methods have leveraged more advanced computational techniques, including deep neural networks[7,8] and specialized hardware [9]. Despite these advancements, faster and more lightweight variant calling methods enable researchers with fewer available resources to identify variation across more samples or leverage variant calls as a quality control technique.

Runtime efficiency and speed are especially important when analyzing large data sets at the cohort or population level. The recent release of 3,202 samples across diverse populations at 30x whole-genome coverage by the 1000 Genomes Project (1KGP), for example, is an important step towards improving variant calling methods [10]. A per-sample SNV caller must scale to population-level variant detection by leveraging features such as genome VCF (gVCF) output and high per-sample variant call accuracy. Furthermore, it must cope with changes in sequencing technologies. As an example, The Trans-Omics for Precision Medicine program (https://www.nhlbiwgs.org/), has sequenced more than 93,000 whole genomes to date and aims to sequence at least 155,000 samples in total. The overall volume and heterogeneity of these data well exceeds those benchmarks that are often used to establish specific methods. The NIST Genome In the Bottle Project (GIAB) has released multiple benchmarks and data sets [11] to provide an objective way to benchmark novel SNV callers [12] and other variant callers [13] on a single trio basis. These benchmarks are curated in part over a trio (HG002, HG003, HG004) and covers the vast majority of the human genome. These benchmarks enabled the improvement of SNV calling over the past years and the establishment of novel variant calling. Considerable efforts have also been focused on increasing the computational efficiency of sequence analysis and variant calling at scale, with GATK[14] , DeepVariant [8], Edico Genome's Dragen[9], and other state-of the art methods leveraging distributed software and hardware-optimized technologies. However, these methods often require commitments to external infrastructure or internal technology development to be applied effectively, which may not be well-suited for rapid turnaround times or cost-effective execution of variant calling at scale.

The ideal variant caller would therefore manage both scalability issues and sources of data heterogeneity. Further, the method should also allow rapid turnaround times for the generation of single-sample call sets so that different parameters can be evaluated. Here, we describe xAtlas, a lightweight and accurate single-sample SNV and small indel variant caller. xAtlas includes features that allow it to easily scale to population-scale sample sets, including support for CRAM[15] and gVCF file formats, minimal computational requirements, and fast runtimes. xAtlas demonstrates high accuracy when compared against GIAB reference benchmarks and favorable performance on large sample sets, such as those from 1KGP. xAltas is implemented

as a command line application written in C++ and is available under an open source BSD license.

Variant calling process

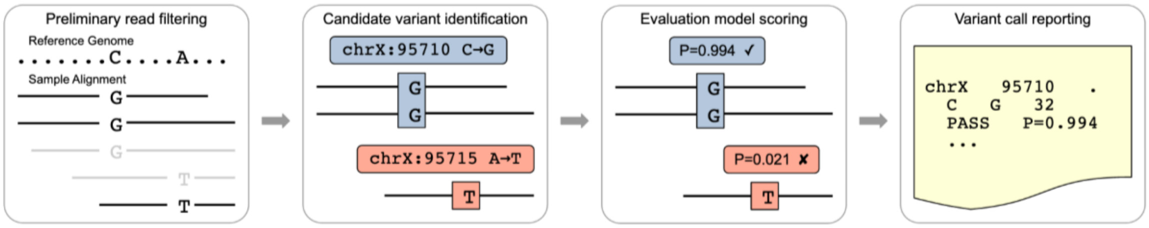

Figure 1*: The major stages of xAtlas variant calling process.*

Figure 1 provides an overview of the main steps of xAtlas. xAtlas variant calling is performed in three stages: preliminary read filtering, collecting candidates for variant calling from aligned reads, and evaluating whether to call or report each candidate variant. First, reads that are marked as unmapped, duplicate reads, or have a mapping quality below a user-configurable minimum threshold are filtered out from further processing. Next, candidate variants are identified by aggregating per-read sequence variations from the reference genome with matching left-aligned genomic coordinates.

While collecting candidate variants to evaluate, xAtlas also records values for a number of sequence and alignment attributes associated with each candidate variant, including mean base quality value, mean alignment quality for reads covering the candidate variant's genomic position, and coverage counts for reads supporting candidate variant allele. These values are provided either to a SNV or to an indel logistic regression model to assign a confidence score of the candidate variant's likelihood of being a real variant. This likelihood score, along with separate cutoffs for low alignment quality and other filtering criteria, determine whether the variant is called and whether a callled variant is filtered. xAtlas allows the user to reconfigure the logistic regression intercepts and variable coefficients of these models. After assigning variant confidence scores from the logistic regression models and applying VCF filters, xAtlas genotypes and reports the candidate variant in the output VCF. SNVs and indels are written to separate output VCF files.

xAtlas' variant evaluation model excludes some techniques that are employed by other single-sample variant callers. Notably, xAtlas does not perform local realignment around candidate variant regions, which contributes in part to reduced application runtimes. xAtlas also attempts to call no more than one SNV or indel variant allele at a given genomic locus. Despite these model limitations, xAtlas nonetheless achieves a high degree of variant sensitivity and precision.

xAtlas is implemented as a command-line application written in C++ that employs HTSlib[16] to handle HTS alignment and variant file formats. Input sample alignment files may be in either

BAM[17] or CRAM [15] format and output variant calls are written in VCF format[18]. xAtlas includes options for multithreading and writing output in genome VCF format (gVCF), in which xAtlas includes additional VCF entries to capture coverage information for regions between called variants.

Variant call assessment with NIST Genome in a Bottle HG002 benchmark data set

xAtlas variant calling accuracy was assessed by measuring the concordance between xAtlas variant calls on reference alignments and corresponding benchmark call sets from release 3.3.2 of the NIST Genome in a Bottle Project (GIAB) [12]. **Table 1** summarizes the results of xAtlas variant calling on the GIAB Ashkenazi trio reference samples. xAtlas variant calls on an HG002 sample at 60x mean whole-genome coverage had a precision of 98.88% and a sensitivity of 99.75% for SNVs and a precision of 95.30% and a sensitivity of 87.21% for indels, relative to SNVs and indels in the GIAB HG002 benchmark call set within high-confidence regions. xAtlas achieved these results in 1.9 hours.

Sample	Coverage	SNV precision	SNV sensitivity	Indel precision	Indel sensitivity
HG002	60.72	0.9888	0.9975	0.9530	0.8721
HG003	53.09	0.9900	0.9976	0.9527	0.8675
HG004	61.47	0.9895	0.9979	0.9526	0.8715

Table 1*: Precision and sensitivity of xAtlas SNV and indels calls on Ashkenazi trio reference samples from NIST GIAB.*

xAtlas performance comparison across NA12878 benchmark data sets

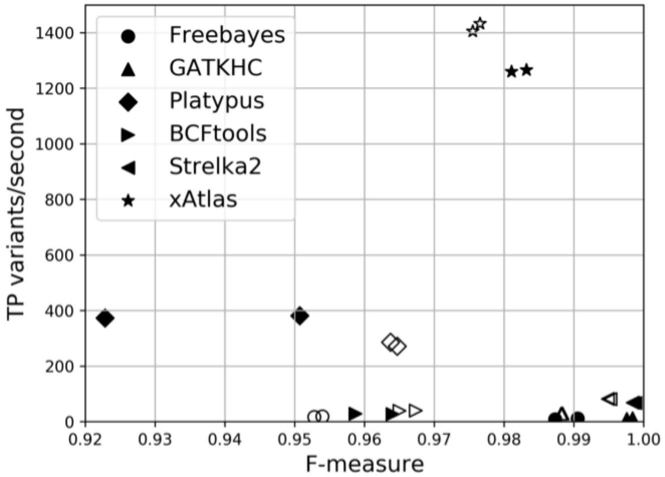

Figure 2*: Per-second rates of called variants concordant to GIAB NA12878 high-confidence variants as true positives per second in relation to F-measure for variants called on two Illumina HiSeq X (open) and NovaSeq (filled) NA12878 whole-genome samples at 30x average coverage.*

Next we assessed xAtlas performance in terms of speed and accuracy across another GIAB benchmark sample NA12878[19]. xAtlas, Freebayes[20], GATK HaplotypeCaller [14], Platypus [21], and Strelka2[22], and a Samtools and BCFtools-based[17] variant calling pipelines were benchmarked on multiple NA12878 Illumina HiSeqX and NovaSeq samples ranging from 20x to 54x average whole-genome coverage. xAtlas achieved variant concordance F-measures from 98.70% to 99.74% for SNVs and from 84.36% to 92.97% for indels. xAtlas demonstrated the highest rate of true positive variants called per second across three whole-genome NA12878 samples at 30x coverage each (**Figure 2,** see **Supplementary Table 1** for details). xAtlas required only 2,865 seconds to call variants on a 54x Illumina NovaSeq sample when running in single threaded mode with a resident memory usage of 3.7 GB. Runtimes among other variant callers measured was followed by Platypus, showing 3-to 4-fold increased runtimes compared to xAtlas, with xAtlas achieving higher variant concordance F-measures for all samples tested (ranging from 0.9227 to 0.9595 for Platypus and from 0.9753 to 0.9793 for xAtlas).

Variant callset generation on 3202 samples from the 1000 Genomes Project

To assess the performance of xAtlas on a large-scale NGS dataset, xAtlas was run on a data set of 3,202 samples from the 1000 Genomes Project [10] (1KGP), with a mean whole-genome coverage of 34.17 across the sample set. For the 1KGP data set, xAtlas was run in single-threaded operation for each sample, in parallel on an HPC cluster, with an average runtime of 1.7 hours per sample and a cumulative runtime of 5437.4 hours for the 3,202 samples. An average of 4.585M passing SNVs and 880.3K passing indels were called per sample, for a total of 17.5B passing variants called across all samples.

The transition vs transversion (Ti/Tv) ratios of passing SNVs called in autosomal chromosomes for the 1KGP data set had a mean of 1.928 with a standard deviation of 0.0094. Within the Ashkenazi trio, when limited to GIAB high-confidence variant calling regions determined for the trio, the average Ti/Tv ratio for passing variants in the Ashkenazi trio is 2.08. This is in agreement with the expectation of a Ti/Tv ratio of 2.07-2.10 for whole-genome sequencing in humans[23]. GIAB high confidence regions for the Ashkenazi trio cover an average of 83.6% of non-masked autosomal sequences in the GRCh38 reference genome.

Principal component analysis of SNVs called across these samples support variant call accuracy across diverse populations. **Figure 3** shows a PCA plot of the first two principal components of samples from the 3,202 sample data set for SNVs called on chromosome 22 with minor allele frequency (MAF) of at least 0.05. Samples within the same or related populations are found in identifiable clusters, while population clusters show proximity to related populations (**Figure 3a**). Population-level clustering is also observed when plotting principal components 1 vs 3 (**Figure 3b**), as well as principal components 2 vs 3 (**Figure 3c**).

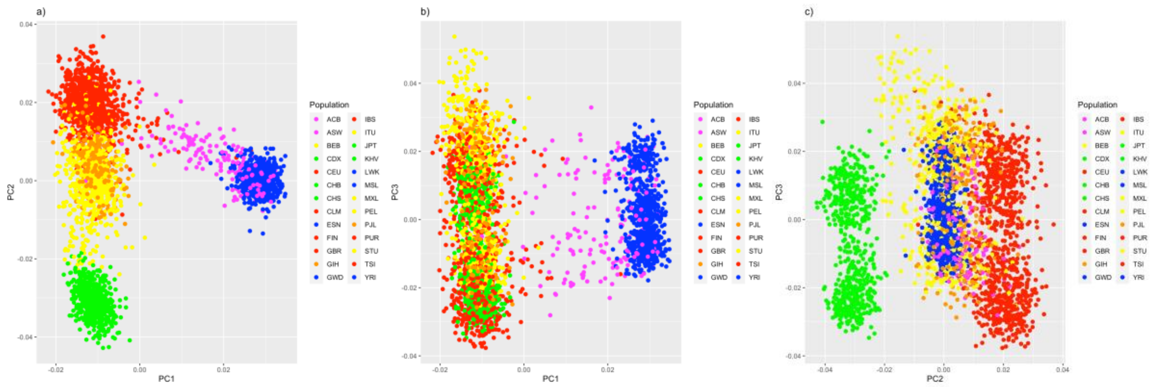

Figure 3: *PCA plots of chromosome 22 SNVs with MAF ≥ 0.05 called by xAtlas on 3202 samples from the 1000 Genomes Project, showing (a) PC1 vs. PC2, (b) PC1 vs. PC3, and (c) PC2 vs. PC3.*

To summarize, xAtlas has demonstrated a combination of computational efficiency and variant call accuracy. Sensitivity and precision rates for both SNVs and indels called by xAtlas rank among those of other variant calling methods that have been used in practice. xAtlas has permitted fast and cost-effective variant analysis across multiple projects at the BCM-HGSC consisting of tens of thousands of whole-genome samples. For small or large-scale variant analysis, xAtlas can be scaled and run in compute environments ranging from a single laptop to large HPC clusters or arrays of cloud instances. With the ability to generate VCFs and gVCF-formatted variant call sets in terms of minutes or hours per sample, development of new variant analysis methods can also be carried out with rapid turnaround rates.

Methods

Variant Call Quality Assessment

xAtlas is a command-line C++ application that employs HTSlib[17] to handle alignment and variant call file formats. Input sample alignment files may be in either BAM [17] or CRAM[15] format. When writing output in VCF format [18], xAtlas may optionally include non-variant VCF entries spanning regions not covered by variants formatted in genome VCF (gVCF) format (https://sites.google.com/site/gvcftools/) to facilitate downstream multi-sample variant analyses. The application also may be built with multithreading support, which allows the processes of reading the input alignment file, processing SNVs, and processing indels to be handled each in a separate thread.

Variant Detection and Evaluation

xAtlas variant calling is performed in the following high-level stages: preliminary read filtering; collecting candidates for variant calls from the alignment file; evaluating each candidate variant;

and reporting candidate variants (**Figure 1**). As reads are scanned from the input alignment, a read will be filtered out from variant calling if it is marked as an unmapped, as a duplicate read, or alternatively has a mapping quality score below a minimum threshold, with a default of one. Candidate sequence variations are then collected from the unfiltered reads and grouped for variant calls. To aggregate candidates, sequence variations are identified within each read by locating the coordinates at which sequences differ from the provided reference genome. The SAM format's CIGAR string, which defines the edit operations between the read's sequence and the reference sequence at its mapped position, is used to determine variant coordinates. SNVs
are defined as point differences between reference and aligned sample sequences within the spans of CIGAR match operators. Variant alleles are assigned reference coordinates that correspond to its parsimonious representation within the alignment, as defined by Tan et al. [24]. While collecting candidate variants to evaluate, xAtlas also records multiple sequence and alignment attributes associated with each candidate variant, including base quality values and coverage ratios for reads supporting the reference sequence vs. those supporting the variant allele. These features are evaluated together to assign a confidence score for the candidate variant, utilizing either a SNV or indel candidate variant evaluation model based on logistic regression. xAtlas allows the user to redefine the logistic regression intercepts and variable coefficients of these models with values that may be derived from retraining on new samples. Thresholds for both confidence scores and candidate variant features determine whether a candidate variant will be called and if a called variant will be filtered in the output VCF file. Other filters may also be applied to candidate variants based on other features not evaluated by the candidate evaluation models, such as if there are too few reads supporting the candidate variant allele.

After assigning variant confidence scores from the logistic regression models and applying filters, xAtlas then determines the most likely genotype and reports the candidate variant in the VCF. A variant call is reported in the VCF only if the candidate's logistic regression value is greater than an adjustable cutoff, with a default value of 0.25. If multiple variants may be reported at the same position, xAtlas reports only the variant at that position with the greatest number of reads supporting the variant sequence. For SNVs, if there are still multiple candidates tied for the greatest number of supporting reads, the candidate variant with the highest logistic regression value is then selected. xAtlas assigns the genotype 1/1, 0/1, or 0/0 to called variants. For indel, genotypes are assigned based on cutoffs for the ratio of reads supporting the variant allele to the total number of reads overlapping the indel. For SNVs, each SNV is assigned the genotype with the highest genotype likelihood as determined by xAtlas.

Retraining Candidate Variant Evaluation Models

The logistic regression model retraining performed as part of this study was performed by building sets of positive and negative examples of variant sites from pairs of sample alignments and using subsets of these variant sites in logistic regression model fitting. The set of all possible candidate variant sites and the values that xAtlas supplies to the SNV and indel logistic regression models were compiled for each sample. Subsets of positive and negative variant site examples were then derived from this set based on variant site overlaps with a truth set of high-

confidence variants and with high-confidence variant regions. Positive variant sites were selected from variant sites present in both technical replicates, overlapping the NIST high-confidence variants, and restricted to the NIST high-confidence regions. Two types of negative variant sample sites were compiled, where variant sites are either present in both technical replicates or present in only one of the two replicates, with both types restricted to the NIST high-confidence regions but not overlapping the NIST high-confidence variants. Each of these comprised half of the negative example variant sites in assembled training and testing sets. Training and testing sets were compiled as non-overlapping sets of 10,000 randomly sampled positive and negative variant site examples, with a 1:1 ratio of positive vs. negative examples in each set. Logistic regression model fitting using these training and testing sets was performed using theLogisticRegression classifier from scikit-learn [25].

xAtlas performance assessment on GIAB and 1000 Genomes Project

All GIAB and 1000 Genomes Project sample alignments used as input for xAtlas were in CRAM format and aligned to the GRCh38 human reference genome. xAtlas runs were performed on a Linux HPC cluster at the BCM-HGSC, with runtimes measured using system time utilities on the HPC cluster. xAtlas command line invocations included the “--gvcf” and “--bgzf” output options.

GIAB benchmark comparisons

xAtlas variant call precision and sensitivity measurements were calculated by the vcfeval function in RTG-tools[26] version 3.9.1. SNVs for HG002, HG003, and HG004 derived from NIST Genome in a Bottle release 3.3.2 were used as truth sets in vcfeval comparisons, with corresponding GIAB high-confidence variant region BED files used as evaluation regions in these comparisons.

1000 Genomes Project data analysis

Principal component analysis was carried out by constructing a project-level VCF (pVCF) file of SNVs called by xAtlas on 3202 samples from the 1000 Genomes Project sample set and estimating principal components based on the presence or absence of passing variants across the sample set at each genetic locus. First, the union of all genetic loci to be considered for principal component estimation was determined. This was defined to be all genetic loci for which xAtlas called a passing SNV on at least one sample with the 3202 sample set, and for which the minor allele frequency (MAF) of the called variant within the 3202 sample set was at least 0.05. Next, a pVCF file was constructed by combining the xAtlas-called genotypes for the 3202 samples into a single file in VCF format. Each VCF record in the pVCF file represents a genetic locus from the previously determined set of all xAtlas-called genetic loci across the sample set, and each of the 3202 samples represented by a sample-level column denoting the presence or absence of an xAtlas-called genotype at each genetic loci. Principal component analysis on the pVCF file was performed by PLINK[27] version 1.9.

Availability of supporting source code and requirements

xAtlas source code and instructions may be downloaded from https://github.com/jfarek/xatlas. HTSlib (https://github.com/samtools/htslib) is required for building xAtlas.

Availability of supporting data

NIST GIAB data was obtained from:
https://ftp-trace.ncbi.nlm.nih.gov/giab/ftp/release/
1000 Genomes Project data was obtained from: https://www.internationalgenome.org/data-portal/data-collection/30x-grch38

Funding

This work has been supported by NHGRI Centers for Common Disease Genomics grant 5UM1HG008898-02.

Conflicts of interest

The authors declare no conflicts of interest.

Authors' contributions

Application development and code implementation: JF, DH. Sample analysis: JF, YZ, AP,AM,OK. Project coordination: WS, RG, ZK, FS. All authors contributed to the manuscript writing.

Acknowledgements

Sample data for the 1000 Genomes Project were generated at the New York Genome Center with funds provided by NHGRI Grant 3UM1HG008901-03S1.

References

1. Wagner J, Olson ND, Harris L, Khan Z, Farek J, Mahmoud M, et al.. Benchmarking challenging small variants with linked and long reads. bioRxiv.

2. Olson ND, Wagner J, McDaniel J, Stephens SH, Westreich ST, Prasanna AG, et al.. precisionFDA Truth Challenge V2: Calling variants from short- and long-reads in difficult-to-map regions. bioRxiv. bioRxiv;

3. Nielsen R, Paul JS, Albrechtsen A, Song YS. Genotype and SNP calling from next-generation sequencing data. *Nat Rev Genet*. 12:443–512011;

4. Altman RB, Prabhu S, Sidow A, Zook JM, Goldfeder R, Litwack D, et al.. A research roadmap for next-generation sequencing informatics. *Sci Transl Med*. 8:335ps102016;

5. Koboldt DC. Best practices for variant calling in clinical sequencing. *Genome Med*.

12:912020;

6. Taliun D, Harris DN, Kessler MD, Carlson J, Szpiech ZA, Torres R, et al.. Sequencing of 53,831 diverse genomes from the NHLBI TOPMed Program. *Nature*. 590:290–92021;

7. Luo R, Sedlazeck FJ, Lam T-W, Schatz MC. A multi-task convolutional deep neural network for variant calling in single molecule sequencing. *Nat Commun*. 10:9982019;

8. Poplin R, Chang P-C, Alexander D, Schwartz S, Colthurst T, Ku A, et al.. A universal SNP and small-indel variant caller using deep neural networks. Nature Biotechnology.

9. Miller NA, Farrow EG, Gibson M, Willig LK, Twist G, Yoo B, et al.. A 26-hour system of highly sensitive whole genome sequencing for emergency management of genetic diseases. *Genome Med*. 7:1002015;

10. Byrska-Bishop M, Evani US, Zhao X, Basile AO, Abel HJ, Regier AA, et al.. High coverage whole genome sequencing of the expanded 1000 Genomes Project cohort including 602 trios. bioRxiv. bioRxiv;

11. Zook JM, Salit M. Genomes in a bottle: creating standard reference materials for genomic variation - why, what and how? Genome Biology.

12. Zook JM, Chapman B, Wang J, Mittelman D, Hofmann O, Hide W, et al.. Integrating human sequence data sets provides a resource of benchmark SNP and indel genotype calls. *Nat Biotechnol*. Nature Publishing Group; 32:246–512014;

13. Zook JM, Hansen NF, Olson ND, Chapman L, Mullikin JC, Xiao C, et al.. A robust benchmark for detection of germline large deletions and insertions. *Nat Biotechnol*. 38:1347–552020;

14. McKenna A, Hanna M, Banks E, Sivachenko A, Cibulskis K, Kernytsky A, et al.. The Genome Analysis Toolkit: a MapReduce framework for analyzing next-generation DNA sequencing data. *Genome Res*. 20:1297–3032010;

15. Fritz MH-Y, Hsi-Yang Fritz M, Leinonen R, Cochrane G, Birney E. Efficient storage of high throughput DNA sequencing data using reference-based compression. Genome Research.

16. Bonfield JK, Marshall J, Danecek P, Li H, Ohan V, Whitwham A, et al.. HTSlib: C library for reading/writing high-throughput sequencing data. *Gigascience*. 2021; doi: 10.1093/gigascience/giab007.

17. Li H, Handsaker B, Wysoker A, Fennell T, Ruan J, Homer N, et al.. The Sequence Alignment/Map format and SAMtools. *Bioinformatics*. 25:2078–92009;

18. Danecek P, Auton A, Abecasis G, Albers CA, Banks E, DePristo MA, et al.. The variant call format and VCFtools. *Bioinformatics*. 27:2156–82011;

19. Zook JM, Catoe D, McDaniel J, Vang L, Spies N, Sidow A, et al.. Extensive sequencing of seven human genomes to characterize benchmark reference materials. Sci Data. p. 160025.

20. Garrison E, Marth G. Haplotype-based variant detection from short-read sequencing. arXiv [q-bio.GN].

21. Rimmer A, Phan H, Mathieson I, Iqbal Z, Twigg SRF, WGS500 Consortium, et al.. Integrating mapping-, assembly- and haplotype-based approaches for calling variants in clinical sequencing applications. *Nat Genet*. 46:912–82014;

22. Kim S, Scheffler K, Halpern AL, Bekritsky MA, Noh E, Källberg M, et al.. Strelka2: fast and accurate calling of germline and somatic variants. *Nat Methods*. 15:591–42018;

23. Liu Q, Guo Y, Li J, Long J, Zhang B, Shyr Y. Steps to ensure accuracy in genotype and SNP calling from Illumina sequencing data. *BMC Genomics*. 13 Suppl 8:S82012;

24. Tan A, Abecasis GR, Kang HM. Unified representation of genetic variants. *Bioinformatics*. 31:2202–42015;

25. Pedregosa F, Cauvet E, Varoquaux G, Pallier C, Thirion B, Gramfort A. Learning to Rank from Medical Imaging Data. Machine Learning in Medical Imaging.

26. Cleary JG, Braithwaite R, Gaastra K, Hilbush BS, Inglis S, Irvine SA, et al.. Comparing Variant Call Files for performance benchmarking of next-generation sequencing variant calling pipelines. bioRxiv.

27. Purcell S, Neale B, Todd-Brown K, Thomas L, Ferreira MAR, Bender D, et al.. PLINK: A Tool Set for Whole-Genome Association and Population-Based Linkage Analyses. The American Journal of Human Genetics.

Click here to access/download
Supplementary Material
Supplementary_Table1.xlsx

Baylor College of Medicine
Human Genome Sequencing Center

One Baylor Plaza
Houston, Texas 77030-3498

TEL: (713)798-6539
FAX: (713)798-5741

August 13th, 2021

Dear Editor,

Please find enclosed a copy of the manuscript “xAtlas: Scalable small variant calling across heterogeneous next-generation sequencing experiments”, which we submit for consideration to *GigaScience*.

In our manuscript, we present a lightweight and highly accurate method to identify SNVs and small indels across short read Illumina data. xAtlas is able to identify SNV with 98.88% precision and 99.75% recall across a 60x HG002 CRAM file in 116 minutes CPU time. xAtlas is regularly used here at Baylor College of Medicine inside the Human Genome Sequencing Center in the production pipeline for sequencing data and analysis. Thus, we believe it is useful also for many other labs or scientists in general.

As you know, the tendency to have ever larger cohorts and thus sample size is ongoing while there are also tendencies to change reference genome (e.g. T2T, ethnicity specific etc) or other parameters or properties across an Next Generation Sequencing analysis. To achieve all this one needs a lightweight SNV caller such as xAtlas. xAtlas is designed to rapidly provide SNV calls (substitutions and indel) with low resource requirements. In addition, xAtlas supports CRAM files as input and gVCF files as output, enabling further applications at scale. Given the speed of xAtlas, it is also possible to utilize the program as a quality control to assess if mapping and other previous steps in the analysis worked correctly.

Taken all together, we are confident that this work will have a high degree of impact, citations and is of interest to the readers of GigaScience. We further suggest potential reviewers based on their experience working on genomic data and involvement in developing methods around SNV calling: Andrew Carrol (Product lead, Google, acarroll.dna@gmail.com), Ruibang Luo (Assistant Professor, University of Hong Kong, rbluo@cs.hku.hk), Bernhard Y. Renard (Group lead Robert Koch Institute, RenardB@rki.de), Yuk Yee Leung (Assistant Professor, University of Pennsylvania, yyee@pennmedicine.upenn.edu), Michael Jantsch (Professor, Medical university of Vienna, michael.jantsch@meduniwien.ac.at)

Warm Regards,

Fritz J. Sedlazeck, Ph.D.

Assistant Professor,
Human Genome Sequencing Center,
Baylor College of Medicine,
Houston, TX, 77030
