# Supplementary material for: xAtlas: scalable small variant calling across heterogeneous next-generation sequencing experiments
Source: Gigascience. 2023 Jan 16;12:giac125. doi: 10.1093/gigascience/giac125 (PMC9841152; doi:10.1093/gigascience/giac125)
Supplement: giac125_GIGA-D-21-00249_Revision_1 [file giac125_giga-d-21-00249_revision_1.pdf]

GigaScience

xAtlas: Scalable small variant calling across heterogeneous next-generation sequencing experiments

--Manuscript Draft--

**Manuscript Number:**	GIGA-D-21-00249R1
**Full Title:**	xAtlas: Scalable small variant calling across heterogeneous next-generation sequencing experiments
**Article Type:**	Technical Note
**Funding Information:**	National Institutes of Health (5UM1HG008898-02) — Dr Richard Gibbs
**Abstract:**	Background: The growing volume and heterogeneity of next-generation sequencing (NGS) data complicate the further optimization of identifying DNA variation, especially considering that curated high-confidence variant call sets frequently used to validate these methods are generally developed from the analysis of comparatively small and homogeneous sample sets.<br><br>Findings: We have developed xAtlas, a single-sample variant caller for single nucleotide variants (SNV) and small insertions and deletions (indels) in NGS data. xAtlas features rapid runtimes, support for CRAM and gVCF file formats, and retraining capabilities. xAtlas reports SNVs with 99.75% recall and 98.88% precision across a reference HG002 sample at 60x whole-genome coverage in less than 2 CPU hours. Applying xAtlas to 3,202 samples at 30x whole-genome coverage from the 1000 Genomes Project achieves an average runtime of 1.7 hours per sample and a clear separation of the individual populations in principal component analysis across called SNVs.<br><br>Conclusions: xAtlas is a fast, lightweight, and accurate SNV and small indel calling method. Source code for xAtlas is available under a BSD 3-clause license at https://github.com/jfarek/xatlas
**Corresponding Author:**	Fritz J Sedlazeck<br>Baylor College of Medicine<br>Houston, UNITED STATES
**Corresponding Author Secondary Information:**	
**Corresponding Author's Institution:**	Baylor College of Medicine
**Corresponding Author's Secondary Institution:**	
**First Author:**	Jesse Farek
**First Author Secondary Information:**	
**Order of Authors:**	Jesse Farek
	Daniel Hughes
	William Salerno
	Yiming Zhu
	Aishwarya Pisupati
	Adam Mansfield
	Olga Krasheninina
	Ginger Metcalf
	Eric Boerwinkle
	Donna Muzny

Powered by Editorial Manager® and ProduXion Manager® from Aries Systems Corporation

	Richard Gibbs
	Ziad Khan
	Fritz Sedlazeck
**Order of Authors Secondary Information:**	
**Response to Reviewers:**	We submitted a detailed response to each of the reviewers comments in the cover letter.
**Additional Information:**	
**Question**	**Response**
Are you submitting this manuscript to a special series or article collection?	No
**Experimental design and statistics**<br><br>Full details of the experimental design and statistical methods used should be given in the Methods section, as detailed in our Minimum Standards Reporting Checklist. Information essential to interpreting the data presented should be made available in the figure legends.<br><br>Have you included all the information requested in your manuscript?	Yes
**Resources**<br><br>A description of all resources used, including antibodies, cell lines, animals and software tools, with enough information to allow them to be uniquely identified, should be included in the Methods section. Authors are strongly encouraged to cite Research Resource Identifiers (RRIDs) for antibodies, model organisms and tools, where possible.<br><br>Have you included the information requested as detailed in our Minimum Standards Reporting Checklist?	Yes
**Availability of data and materials**<br><br>All datasets and code on which the conclusions of the paper rely must be either included in your submission or	Yes

Powered by Editorial Manager® and ProduXion Manager® from Aries Systems Corporation

deposited in publicly available repositories (where available and ethically appropriate), referencing such data using a unique identifier in the references and in the "Availability of Data and Materials" section of your manuscript.<br><br>Have you have met the above requirement as detailed in our Minimum Standards Reporting Checklist?	

Manuscript
Click here to access/download;Manuscript;Manuscript.docx

xAtlas: Scalable small variant calling across heterogeneous next-generation sequencing experiments

Jesse Farek[1,*], Daniel Hughes[1,2,*], William Salerno[3,1], Yiming Zhu[1], Aishwarya Pisupati[1], Adam Mansfield[3,1], Olga Krasheninina[3,1], Ginger Metcalf[1], Eric Boerwinkle[1,4], Donna M Muzny[1], Richard Gibbs[1], Ziad Khan[1], Fritz J. Sedlazeck[1]

[1] Baylor College of Medicine Human Genome Sequencing Center, Houston, Texas, 77030
[2] Columbia University, New York, New York, 10027
[3] Regeneron Pharmaceuticals, Inc., Tarrytown, NY, 10591
[4] Human Genetics Center, The University of Texas Health Science Center at Houston, Houston, Texas, 77030,USA.

*: equal contribution
Corresponding: Jesse.Farek@bcm.edu, fritz.sedlazeck@bcm.edu

Abstract

Background: The growing volume and heterogeneity of next-generation sequencing (NGS) data complicate the further optimization of identifying DNA variation, especially considering that curated high-confidence variant call sets frequently used to validate these methods are generally developed from the analysis of comparatively small and homogeneous sample sets.

Findings: We have developed xAtlas, a single-sample variant caller for single nucleotide variants (SNV) and small insertions and deletions (indels) in NGS data. xAtlas features rapid runtimes, support for CRAM and gVCF file formats, and retraining capabilities. xAtlas reports SNVs with 99.11% recall and 98.43% precision across a reference HG002 sample at 60x whole-genome coverage in less than 2 CPU hours. Applying xAtlas to 3,202 samples at 30x whole-genome coverage from the 1000 Genomes Project achieves an average runtime of 1.7 hours per sample and a clear separation of the individual populations in principal component analysis across called SNVs.

Conclusions: xAtlas is a fast, lightweight, and accurate SNV and small indel calling method. Source code for xAtlas is available under a BSD 3-clause license at https://github.com/jfarek/xatlas.

Findings

Over the past 20 years multiple methods and approaches have surfaced, which aim to identify small variants across next-generation sequencing (NGS) short read data [1–3]. The improvement of methods to identify single nucleotide variants (SNVs) and small insertions and deletions (indels) from NGS data remains an active area of research [1,2]. Currently, small

variant calling methods exceed sensitivity and precision of 99% for well-characterized samples [4]. There are ongoing efforts to refine small variant calls to address new demands in research and clinical domains, such as the need for reliably accurate and reproducible variant calls in clinical settings [5] and characterizing rare variants implicated in common diseases [6]. Considerable efforts have also been focused on increasing the computational efficiency of variant calling at scale. As with other secondary genomic analyses, recent small variant calling methods have leveraged more advanced computational techniques, including deep neural networks [7,8] and specialized hardware [9]. Nevertheless, these advancements often come with demands for more compute or specialized hardware. Thus, faster and more lightweight variant calling methods are still desirable as they enable researchers with fewer available resources to identify variation across more samples or leverage variant calls as a quality control technique.

Runtime efficiency and speed are especially important when analyzing large data sets at the cohort or population level. The recent release of 3,202 samples across diverse populations at 30x whole-genome coverage by the 1000 Genomes Project (1KGP), for example, is an important step towards improving variant calling methods [10]. A per-sample SNV caller must scale to population-level variant detection by leveraging features such as genome VCF (gVCF) output and high per-sample variant call accuracy. Furthermore, it must cope with changes in sequencing technologies. As an example, The Trans-Omics for Precision Medicine program (https://www.nhlbiwgs.org/), has sequenced more than 93,000 whole genomes to date and aims to sequence at least 155,000 samples in total. The overall volume and heterogeneity of these data well exceed those benchmarks that are often used to establish specific methods. The NIST Genome In the Bottle Project (GIAB) has released multiple benchmarks and data sets [11] to provide an objective way to benchmark novel SNV callers [12] and other variant callers [13] on a single trio basis. These benchmarks are curated in part over a trio (HG002, HG003, HG004) and covers the vast majority of the human genome. These benchmarks enabled the improvement of SNV calling over the past years and the establishment of novel variant calling. Considerable efforts have also been focused on increasing the computational efficiency of sequence analysis and variant calling at scale, with GATK [14] , DeepVariant [8], Illumina Dragen [9], and other state-of the art methods leveraging distributed software and hardware-optimized technologies. However, these methods often require commitments to external infrastructure or internal technology development to be applied effectively, which may not be well-suited for rapid turnaround times or cost-effective execution of variant calling at scale. The ideal variant caller should therefore allow fast performance and scalability on commodity computing hardware.

Here, we describe xAtlas, a lightweight and accurate single-sample SNV and small indel variant caller. xAtlas includes features that allow it to easily scale to population-scale sample sets, including support for CRAM [15] and gVCF file formats, minimal computational requirements, and fast runtimes. xAtlas demonstrates high accuracy when compared against GIAB reference benchmarks and favorable performance on large sample sets, such as those from 1KGP. xAltas is implemented as a command line application written in C++ and is available under an open source BSD license.

Variant calling process

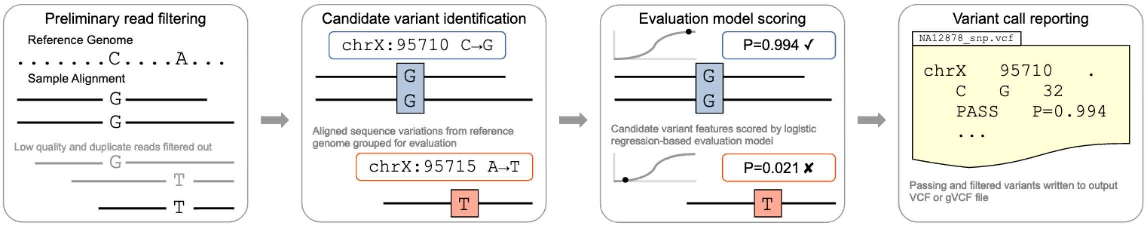

Figure 1*: The major stages of xAtlas variant calling process.*

Figure 1 provides an overview of the main steps of xAtlas. xAtlas variant calling is performed in three stages: preliminary read filtering, collecting candidates for variant calling from aligned reads, and evaluating whether to call or report each candidate variant. First, reads that are marked as unmapped, duplicate reads, or have a mapping quality below a user-configurable minimum threshold are filtered out from further processing. Next, candidate variants are identified by aggregating per-read sequence variations from the reference genome with matching left-aligned genomic coordinates.

While collecting candidate variants to evaluate, xAtlas also records values for a number of sequence and alignment features associated with each candidate variant, including mean base quality value, mean alignment quality for reads covering the candidate variant's genomic position, and coverage counts for reads supporting candidate variant allele. These values are provided either to a SNV or to an indel logistic regression model to assign a confidence score of the candidate variant's likelihood of being a real variant. This likelihood score, along with separate cutoffs for low alignment quality and other filtering criteria, determines whether the variant is called and whether a callled variant is filtered. xAtlas allows the user to reconfigure the logistic regression intercepts and variable coefficients of these models. After assigning variant confidence scores from the logistic regression models and applying VCF filters, xAtlas genotypes and reports the candidate variant in the output VCF. SNVs and indels are written to separate output VCF files.

xAtlas' variant evaluation model excludes some techniques that are employed by other single-sample variant callers. Notably, xAtlas does not perform local realignment around candidate variant regions, which contributes in part to reduced application runtimes. xAtlas also attempts to call no more than one SNV or indel variant allele at a given genomic locus. Despite these model limitations, xAtlas nonetheless achieves a high degree of variant sensitivity and precision.

xAtlas is implemented as a command-line application written in C++ that employs HTSlib [16] to handle HTS alignment and variant file formats. Input sample alignment files may be in either BAM [17] or CRAM [15] format and output variant calls are written in VCF format [18]. xAtlas includes options for multithreading and writing output in genome VCF format (gVCF), in which

xAtlas includes additional VCF entries to capture coverage information for regions between called variants.

Variant call assessment with NIST Genome in a Bottle HG002 benchmark data set

xAtlas variant calling accuracy was assessed by measuring the concordance between xAtlas variant calls on reference alignments and corresponding benchmark call sets from release 4.2.1 of the NIST Genome in a Bottle Project (GIAB) [12]. **Table 1a** summarizes the results of xAtlas variant calling on the GIAB Ashkenazi trio reference samples. xAtlas variant calls on an HG002 sample at 60x mean whole-genome coverage had a precision of 98.43% and a sensitivity of 99.11% for SNVs and a precision of 94.85% and a sensitivity of 86.28% for indels, relative to SNVs and indels in the GIAB HG002 benchmark call set within high-confidence regions. xAtlas achieved these results in 1.9 hours.

**Sample**	**Coverage**	**SNV precision**	**SNV sensitivity**	**Indel precision**	**Indel sensitivity**
HG002	60.72	0.9843	0.9911	0.9485	0.8628
HG003	53.09	0.9855	0.9909	0.9489	0.8603
HG004	61.47	0.9854	0.9911	0.9491	0.8645

Table 1: *Precision and sensitivity of xAtlas SNV and indels calls on Ashkenazi trio reference samples from NIST GIAB relative to GIAB v4.2.1 benchmark small variants.*

xAtlas performance comparison across NA12878 benchmark data sets

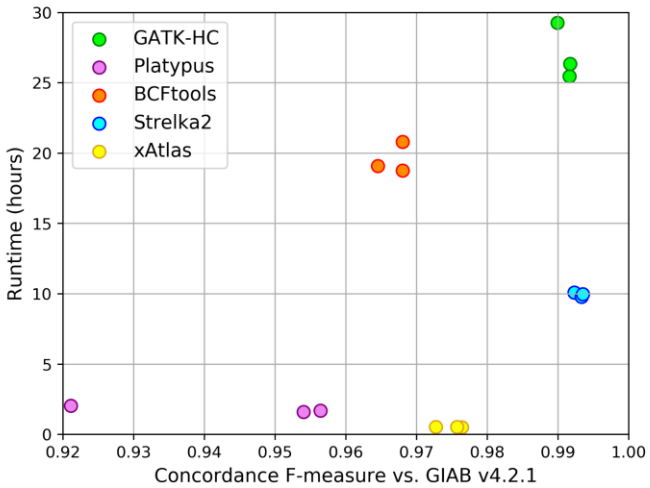

Figure 2: *Variant caller runtimes in single-threaded operation in relation to F-measures of called variants concordant to GIAB v4.2.1 NA12878 high-confidence variants for three Illumina HiSeq X NA12878 whole-genome samples at 30x average coverage. Freebayes had runtimes of between 55.7 and 88.7 hours and is not shown.*

Next, we assessed xAtlas performance in terms of speed and accuracy across another GIAB benchmark sample, NA12878 [20]. xAtlas, Freebayes [21], GATK HaplotypeCaller [14], Platypus [22], and Strelka2 [23], and a Samtools and BCFtools-based [17] variant calling pipelines were benchmarked on three whole-genome NA12878 samples sequenced on the Illumina HiSeqX platform at 30x average whole-genome coverage. xAtlas demonstrated the lowest runtimes of between 0.510 and 0.543 hours across the three NA12878 samples with concordance F-measures of between 0.9727 and 0.9764 when compared to GIAB v4.2.1 benchmark small variants in high confidence regions (**Figure 2**). xAtlas required only 2,865 seconds to call variants on a 54x Illumina NovaSeq sample when running in single threaded mode with a resident memory usage of 3.7 GB. Runtimes among other variant callers measured was followed by Platypus, showing 3-to 4-fold increased runtimes compared to xAtlas, with xAtlas achieving higher variant concordance F-measures for all samples tested relative to Platypus.

Variant callset generation on 3202 samples from the 1000 Genomes Project

To assess the performance of xAtlas on a large-scale NGS dataset, xAtlas was run on a data set of 3,202 samples from the 1000 Genomes Project [10] (1KGP), with a mean whole-genome coverage of 34.17 across the sample set. For the 1KGP data set, xAtlas was run in single-threaded operation for each sample, in parallel on an HPC cluster, with an average runtime of 1.7 hours per sample and a cumulative runtime of 5437.4 hours for the 3,202 samples. An average of 4.585M passing SNVs and 880.3K passing indels were called per sample, for a total of 17.5B passing variants called across all samples.

The transition vs transversion (Ti/Tv) ratios of passing SNVs called in autosomal chromosomes for the 1KGP data set had a mean of 1.928 with a standard deviation of 0.0094. Within the Ashkenazi trio, when limited to GIAB high-confidence variant calling regions determined for the trio, the average Ti/Tv ratio for passing variants in the Ashkenazi trio is 2.08. This is in agreement with the expectation of a Ti/Tv ratio of 2.07-2.10 for whole-genome sequencing in humans [24]. GIAB high confidence regions for the Ashkenazi trio cover an average of 83.6% of non-masked autosomal sequences in the GRCh38 reference genome.

Principal component analysis of SNVs called across these samples support variant call accuracy across diverse populations. **Figure 3** shows a PCA plot of the first two principal components of samples from the 3,202 sample data set for SNVs called on chromosome 22 with minor allele frequency (MAF) of at least 0.05. Samples within the same or related populations are found in identifiable clusters, while population clusters show proximity to related populations (**Figure 3a**). Population-level clustering is also observed when plotting principal components 1 vs 3 (**Figure 3b**), as well as principal components 2 vs 3 (**Figure 3c**).

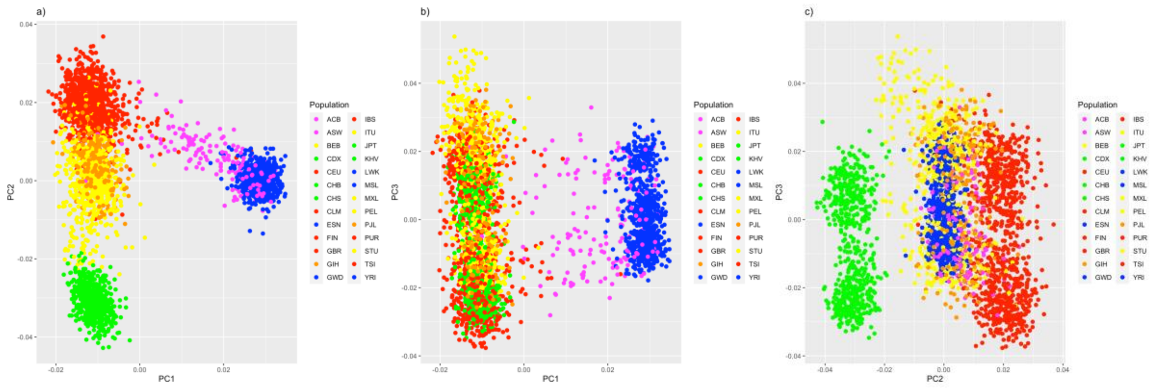

Figure 3: *PCA plots of chromosome 22 SNVs with MAF ≥ 0.05 called by xAtlas on 3202 samples from the 1000 Genomes Project, showing (a) PC1 vs. PC2, (b) PC1 vs. PC3, and (c) PC2 vs. PC3.*

To summarize, xAtlas has demonstrated a combination of computational efficiency and variant call accuracy. Sensitivity and precision rates for both SNVs and indels called by xAtlas rank among those of other variant calling methods that have been used in practice. xAtlas has permitted fast and cost-effective variant analysis across multiple projects at the BCM-HGSC consisting of tens of thousands of whole-genome samples. For small or large-scale variant analysis, xAtlas can be scaled and run in compute environments ranging from a single laptop to large HPC clusters or arrays of cloud instances. With the ability to generate VCFs and gVCF-formatted variant call sets in terms of minutes or hours per sample, development of new variant analysis methods can also be carried out with rapid turnaround rates.

Methods

Variant Call Quality Assessment

xAtlas is a command-line C++ application that employs HTSlib [17] to handle alignment and variant call file formats. Input sample alignment files may be in either BAM [17] or CRAM [15] format. When writing output in VCF format [18], xAtlas may optionally include non-variant VCF entries spanning regions not covered by variants formatted in genome VCF (gVCF) format (https://sites.google.com/site/gvcftools/) to facilitate downstream multi-sample variant analyses. The application also may be built with multithreading support, which allows the processes of reading the input alignment file, processing SNVs, and processing indels to be handled each in a separate thread.

Variant Detection and Evaluation

xAtlas variant calling is performed in the following high-level stages: preliminary read filtering; collecting candidates for variant calls from the alignment file; evaluating each candidate variant; and reporting candidate variants (**Figure 1**).

Preliminary read filtering is performed to filter out uninformative reads. As reads are scanned from the input alignment, reads marked as unmapped, as duplicate reads, or having a mapping quality score below a minimum threshold, with a default of one, are filtered out from further evaluation.

Candidate sequence variations are then collected from the unfiltered reads and grouped for variant calls. To aggregate candidates, sequence variations are identified within each read by locating the coordinates at which sequences differ from the provided reference genome. The SAM format's CIGAR string, which defines the edit operations between the read's sequence and the reference sequence at its mapped position, is used to determine variant coordinates. SNVs are defined as point differences between reference and aligned sample sequences within the spans of CIGAR match operators. Variant alleles are assigned reference coordinates that correspond to its parsimonious representation within the alignment, as defined by Tan et al. [25].

While collecting candidate variants to evaluate, xAtlas also records a number of values associated with sequence and alignment features, such as average base quality score across supporting reads, for each candidate variant. These values are then fed to one of two logistic regression models, for either SNVs or indels, to calculate the probability that the candidate variant is a real variant based on the assessed features. **Table 2** shows the features assessed for each candidate variant and the default model parameters assigned to these features. Thresholds for both confidence scores derived from logistic regression probabilities and specific features determine whether a candidate variant will be called and if a called variant will be called as a filtered variant in the output VCF file. Other filters may also be applied to candidate variants based on other features not evaluated by the candidate evaluation models, such as if there are too few reads supporting the candidate variant allele. xAtlas allows the user to redefine the logistic regression intercepts and variable coefficients of these models with values that may be derived from retraining on new samples.

**(a)** SNV logistic regression model		
**Parameter**	**Default coefficient**	**Description**
`intercept`	-6.6404	Logistic regression intercept
`ratio_score`	11.1192	Score of ratio of reads supporting the candidate SNV vs. all reads covering the SNV
`base_qual`	0.25579	Average base quality at candidate SNV position across supporting reads
`mean_avnqs`	0.12896	Average base quality within 5 bases of candidate SNV position across supporting reads

rel_pos	0.69106	Relative position of the candidate SNV within each read averaged across all supporting reads
titv	0.48511	SNV is transition (1) or transversion (0)

**(b)** Indel logistic regression model		
**Parameter**	**Default coefficient**	**Description**
intercept	-7.1085	Logistic regression intercept
ratio_score	6.22804	Score of ratio of reads supporting the candidate indel vs. all reads covering the indel
strand_dir	2.21407	Indel is supported by reads on both positive and negative strands (true: 1; false: 0)
mean_avnqs	0.07777	Average base quality within 5 bases of candidate indel position across supporting reads
seq_entropy	0.1479	Insertion or deletion sequence entropy calculation
mean_var_rate	-2.13305	Count of variant sequences in each read averaged across all supporting reads

***Table 2**: The default values set by xAtlas for the SNV (a) and indel (b) logistic regression model parameters for scoring candidate variants.*

After assigning confidence scores to candidate variants and applying filters, xAtlas then determines the most likely genotype and reports the candidate variant in the VCF. A variant call is reported in the VCF only if the candidate's logistic regression value is greater than an adjustable cutoff, with a default value of 0.25. If multiple variants may be reported at the same position, xAtlas reports only the variant at that position with the greatest number of reads supporting the variant sequence. For SNVs, if there are still multiple candidates tied for the greatest number of supporting reads, the candidate variant with the highest logistic regression value is then selected. xAtlas assigns the genotype 1/1, 0/1, or 0/0 to called variants. For indel, genotypes are assigned based on cutoffs for the ratio of reads supporting the variant allele to the total number of reads overlapping the indel. For SNVs, each SNV is assigned the genotype with the highest genotype likelihood as determined by xAtlas.

Retraining Candidate Variant Evaluation Models

The logistic regression model retraining performed as part of this study was performed by building sets of positive and negative examples of variant sites from pairs of sample alignments and using subsets of these variant sites in logistic regression model fitting. The set of all possible candidate variant sites and the values that xAtlas supplies to the SNV and indel logistic regression models were compiled for each sample. Subsets of positive and negative variant site examples were then derived from this set based on variant site overlaps with a truth set of high-confidence variants and with high-confidence variant regions. Positive variant sites were selected from variant sites present in both technical replicates, overlapping the NIST high-

confidence variants, and restricted to the NIST high-confidence regions. Two types of negative variant sample sites were compiled, where variant sites are either present in both technical replicates or present in only one of the two replicates, with both types restricted to the NIST high-confidence regions but not overlapping the NIST high-confidence variants. Each of these comprised half of the negative example variant sites in assembled training and testing sets. Training and testing sets were compiled as non-overlapping sets of 10,000 randomly sampled positive and negative variant site examples, with a 1:1 ratio of positive vs. negative examples in each set. Logistic regression model fitting using these training and testing sets was performed using the LogisticRegression classifier from scikit-learn [26].

xAtlas performance assessment on GIAB and 1000 Genomes Project

All GIAB and 1000 Genomes Project sample alignments used as input for xAtlas were in CRAM format and aligned to the GRCh38 human reference genome. xAtlas runs were performed on a Linux HPC cluster at the BCM-HGSC, with runtimes measured using system time utilities on the HPC cluster. xAtlas command line invocations included the “--gvcf” and “--bgzf” output options.

GIAB benchmark comparisons

xAtlas variant call precision and sensitivity measurements were calculated by the vcfeval function in RTG-tools [27] version 3.9.1. SNVs for HG002, HG003, and HG004 derived from NIST Genome in a Bottle release 4.2.1 were used as truth sets in vcfeval comparisons, with corresponding GIAB high-confidence variant region BED files used as evaluation regions in these comparisons.

1000 Genomes Project data analysis

Principal component analysis was carried out by constructing a project-level VCF (pVCF) file of SNVs called by xAtlas on 3202 samples from the 1000 Genomes Project (1000GP) sample set and estimating principal components based on the called genotype of passing variants across the sample set at each genetic locus. Principal component analysis on the final pVCF was performed by PLINK [28] version 1.9.

The project-level VCF for the 3202 1000GP samples was constructed in a few phases. First, the union of all genetic loci to be considered for principal component estimation was determined. This was defined to be all genetic loci for which xAtlas called a passing SNV on at least one sample with the 3202 sample set, and for which the minor allele frequency (MAF) of the called variant within the 3202 sample set was at least 0.05. Next, a pVCF file was constructed by combining the xAtlas-called genotypes for the 3202 samples into a single file in VCF format. Each VCF record in the pVCF file represents a genetic locus from the previously determined set of all xAtlas-called genetic loci across the sample set. Each of the 3202 samples is represented in the pVCF by a sample-level column containing xAtlas-called genotypes from the sample-level VCF across all genetic loci recorded in the pVCF. While this pVCF was constructed using

purpose-built scripts, other utilities that can also be used to construct pVCF files include GLnexus [29], which includes support for gVCFs created by xAtlas.

Availability of supporting source code and requirements

xAtlas source code and instructions may be downloaded from https://github.com/jfarek/xatlas. HTSlib (https://github.com/samtools/htslib) is required for building xAtlas.

Availability of supporting data

NIST GIAB data was obtained from:
https://ftp-trace.ncbi.nlm.nih.gov/giab/ftp/release/
1000 Genomes Project data was obtained from: https://www.internationalgenome.org/data-portal/data-collection/30x-grch38

Funding

This work has been supported by NHGRI Centers for Common Disease Genomics grant 5UM1HG008898-02.

Conflicts of interest

The authors declare no conflicts of interest.

Authors' contributions

Application development and code implementation: JF, DH. Sample analysis: JF, YZ, AP, AM, OK. Project coordination: WS, RG, ZK, FS. All authors contributed to the manuscript writing.

Acknowledgements

Sample data for the 1000 Genomes Project were generated at the New York Genome Center with funds provided by NHGRI Grant 3UM1HG008901-03S1.

References

1. Wagner J, Olson ND, Harris L, Khan Z, Farek J, Mahmoud M, et al.. Benchmarking challenging small variants with linked and long reads. bioRxiv.

2. Olson ND, Wagner J, McDaniel J, Stephens SH, Westreich ST, Prasanna AG, et al.. precisionFDA Truth Challenge V2: Calling variants from short- and long-reads in difficult-to-map regions. bioRxiv. bioRxiv;

3. Nielsen R, Paul JS, Albrechtsen A, Song YS. Genotype and SNP calling from next-generation sequencing data. *Nat Rev Genet*. 12:443–512011;

4. Altman RB, Prabhu S, Sidow A, Zook JM, Goldfeder R, Litwack D, et al.. A research roadmap for next-generation sequencing informatics. *Sci Transl Med*. 8:335ps102016;

5. Koboldt DC. Best practices for variant calling in clinical sequencing. *Genome Med*. 12:912020;

6. Taliun D, Harris DN, Kessler MD, Carlson J, Szpiech ZA, Torres R, et al.. Sequencing of 53,831 diverse genomes from the NHLBI TOPMed Program. *Nature*. 590:290–92021;

7. Luo R, Sedlazeck FJ, Lam T-W, Schatz MC. A multi-task convolutional deep neural network for variant calling in single molecule sequencing. *Nat Commun*. 10:9982019;

8. Poplin R, Chang P-C, Alexander D, Schwartz S, Colthurst T, Ku A, et al.. A universal SNP and small-indel variant caller using deep neural networks. Nature Biotechnology.

9. Miller NA, Farrow EG, Gibson M, Willig LK, Twist G, Yoo B, et al.. A 26-hour system of highly sensitive whole genome sequencing for emergency management of genetic diseases. *Genome Med*. 7:1002015;

10. Byrska-Bishop M, Evani US, Zhao X, Basile AO, Abel HJ, Regier AA, et al.. High coverage whole genome sequencing of the expanded 1000 Genomes Project cohort including 602 trios. bioRxiv. bioRxiv;

11. Zook JM, Salit M. Genomes in a bottle: creating standard reference materials for genomic variation - why, what and how? Genome Biology.

12. Zook JM, Chapman B, Wang J, Mittelman D, Hofmann O, Hide W, et al.. Integrating human sequence data sets provides a resource of benchmark SNP and indel genotype calls. *Nat Biotechnol*. Nature Publishing Group; 32:246–512014;

13. Zook JM, Hansen NF, Olson ND, Chapman L, Mullikin JC, Xiao C, et al.. A robust benchmark for detection of germline large deletions and insertions. *Nat Biotechnol*. 38:1347–552020;

14. McKenna A, Hanna M, Banks E, Sivachenko A, Cibulskis K, Kernytsky A, et al.. The Genome Analysis Toolkit: a MapReduce framework for analyzing next-generation DNA sequencing data. *Genome Res*. 20:1297–3032010;

15. Fritz MH-Y, Hsi-Yang Fritz M, Leinonen R, Cochrane G, Birney E. Efficient storage of high throughput DNA sequencing data using reference-based compression. Genome Research.

16. Bonfield JK, Marshall J, Danecek P, Li H, Ohan V, Whitwham A, et al.. HTSlib: C library for reading/writing high-throughput sequencing data. *Gigascience*. 2021; doi: 10.1093/gigascience/giab007.

17. Li H, Handsaker B, Wysoker A, Fennell T, Ruan J, Homer N, et al.. The Sequence Alignment/Map format and SAMtools. *Bioinformatics*. 25:2078–92009;

18. Danecek P, Auton A, Abecasis G, Albers CA, Banks E, DePristo MA, et al.. The variant call format and VCFtools. *Bioinformatics*. 27:2156–82011;

19*. Olson, ND, Wagner J, McDaniel J, Stephens SH, Westreich ST, Prasanna AG, et al. precisionFDA Truth Challenge V2: Calling variants from short-and long-reads in difficult-to-map

regions. bioRxiv.

20. Zook JM, Catoe D, McDaniel J, Vang L, Spies N, Sidow A, et al.. Extensive sequencing of seven human genomes to characterize benchmark reference materials. Sci Data. p. 160025.

21. Garrison E, Marth G. Haplotype-based variant detection from short-read sequencing. arXiv [q-bio.GN].

22. Rimmer A, Phan H, Mathieson I, Iqbal Z, Twigg SRF, WGS500 Consortium, et al.. Integrating mapping-, assembly- and haplotype-based approaches for calling variants in clinical sequencing applications. *Nat Genet*. 46:912–82014;

23. Kim S, Scheffler K, Halpern AL, Bekritsky MA, Noh E, Källberg M, et al.. Strelka2: fast and accurate calling of germline and somatic variants. *Nat Methods*. 15:591–42018;

24. Liu Q, Guo Y, Li J, Long J, Zhang B, Shyr Y. Steps to ensure accuracy in genotype and SNP calling from Illumina sequencing data. *BMC Genomics*. 13 Suppl 8:S82012;

25. Tan A, Abecasis GR, Kang HM. Unified representation of genetic variants. *Bioinformatics*. 31:2202–42015;

26. Pedregosa F, Cauvet E, Varoquaux G, Pallier C, Thirion B, Gramfort A. Learning to Rank from Medical Imaging Data. Machine Learning in Medical Imaging.

27. Cleary JG, Braithwaite R, Gaastra K, Hilbush BS, Inglis S, Irvine SA, et al.. Comparing Variant Call Files for performance benchmarking of next-generation sequencing variant calling pipelines. bioRxiv.

28. Purcell S, Neale B, Todd-Brown K, Thomas L, Ferreira MAR, Bender D, et al.. PLINK: A Tool Set for Whole-Genome Association and Population-Based Linkage Analyses. The American Journal of Human Genetics.

29. Michael L, Ohad R, John P, Xiaodong B, Jeffrey R, Olga K, et al.. GLnexus: joint variant calling for large cohort sequencing.

We would like to thank the reviewers and the editor for their constructive feedback. For this resubmission, we made essential changes to the benchmarks, extended the method description, and improved the documentation of xAtlas. Overall improved the manuscript and we highlight appreciate this. Furthermore, it was great to see that reviewers tested xAtlas on their own and didn't have any issues running it on local data.

In the following, we reply to each point of the reviewer's questions and we made the changes to the manuscript as well. To improve the visibility we highlighted these changes and responses in blue.

Reviewer #1: In this paper, the authors proposed xAtlas, an open-source NGS variant caller. xAtlas is a fast and lightweight caller with comparable performance with other benchmarked callers. The benchmark comparison in multiple popular short-read platforms (Illumina HiSeq X and NovaSeq) demonstrated xAtlas's capacity to identify small variants rapidly with desirable performance. Although xAtlas is limited to call multi-allelic variants, the high sensitivity (~99.75% recall for ~60x benchmarking datasets) and desirable runtime (<2 hours) enable xAtlas to rapidly filter candidates and be considered as important quality control for further utilization.

The authors presented a detailed explanation of xAtlas's workflow, design decisions and have done complete experiments in benchmarking, while there are still some points the authors need to discuss further listed as follow:

1. The authors reported the performance in multiple coverages of the HG001 sample and the benchmarking result of HG002-4 samples by measuring the concordance with the GIAB truth set (v3.3.2). I noticed that GIAB had updated the GIAB truth sets from v3.3.2 to v4.2.1 for the Ashkenazi trio. The updated version included more difficult regions like segmental duplications and the Major Histocompatibility Complex (MHC) to identify previously unknown clinically relevant variants. Therefore, it would be helpful if the author could give a performance evaluation using the updated truth sets to give a more comprehensive performance of the proposed caller.

   We thank the reviewer for pointing this out. We have now switched to the new benchmark and the results remained largely the same, that xAtlas is fast and remains a high accuracy.

2. In the Methods section, The authors stated the main three stages of the xAtlas variant calling process: read prepossessing, candidates identification, and candidates evaluation. The author fed hand-craft features (base quality, coverages, reference and alternative allele support, etc.) into a logistic regression model to classify true variants and reference calls in the candidate evaluation stage. But in Figure 1, the main workflow of xAtlas, only model scoring was shown, and the evaluation details were not visible. It would be useful if the authors could enrich Figure 1 to add more details to ensure consistency with Methods and facilitate reader understanding.

We thank the reviewer for this suggestion and made the adoption to Figure1. We further now included Table 2 with specific explanations and extended the method section.

3. In Figure 2, the authors reported the xAtlas performance comparison across in HG001 dataset with other variant callers. I noticed that the x-axis was F1-score while the y-axis was true positives per second. The tendency measurement of two metrics seems irrelevant, which might confuse the readers. We suggest the authors make separate

comparisons for the two metrics. (For instance, plot Precision-Recall curves for F1-score measurement and Runtime comparison of various variant callers for speed benchmarking).

We have made the suggested change. The initial figure was so that the fastest method was on top, now its inverted, but the overall conclusion remains. The figure has also been updated to the GIAB benchmark v4.2.1 as suggested.

Zheng, Zhenxian on behalf of the primary reviewer

Reviewer #2: The manuscript describes a variant caller called xAtlas, which uses a logistic regression model to call SNPs after building an alignment and pileup of the reads. The manuscript is clear. The software is built with the aim of being faster than other solutions. However, I have some concerns relative to the method and the manuscript.

1. Unfortunately, the biggest issue with this work is that the gain of speed is obtained with an important sacrifice in accuracy, specially to call indels. I ran xAtlas with two different benchmark datasets and the accuracy, especially for indels and other complex regions was about 20% lower compared to other solutions. Although the difference was smaller, xAtlas is also less accurate than other software tools for SNV calling. It is well known that even a simple SNV caller can achieve high sensitivity and specificity (see results from https://urldefense.proofpoint.com/v2/url?u=https-3A__doi.org_10.1101_gr.107524.110&d=DwIGaQ&c=ZQs-KZ8oxEw0p81sqgiaRA&r=hkDaFIlMMg5o5xD5aQ2Txzhw0BLHnUrK9Cxqzcl0pbM&m=EKbrYWb9s4S-H0LibUFSZHzVZUQl8HR8RNffFrgtXGQ&s=J6hXyOS8tWanQWJ3i5FVj-OmvAhj6RoKYWwr3TEeUKA&e=). However, several SNV errors can be generated by incorrect alignment of reads around indels and other complex regions. For that reason most of the work on variant detection is focused on mechanisms to perform indel realignment or de-novo mini assembly to increase accuracy of both SNV and indel detection. The paper of Strelka is a great example of this (https://urldefense.proofpoint.com/v2/url?u=https-3A__doi.org_10.1038_s41592-2D018-2D0051-2Dx&d=DwIGaQ&c=ZQs-KZ8oxEw0p81sqgiaRA&r=hkDaFIlMMg5o5xD5aQ2Txzhw0BLHnUrK9Cxqzcl0pbM&m=EKbrYWb9s4S-H0LibUFSZHzVZUQl8HR8RNffFrgtXGQ&s=oTSKsfkNs7fm6d27phjYFsdqpNl7Qo2QJJ7nKGT-3To&e=). The

manuscript does not mention if any procedure has been implemented to realign reads or to increase in some way the accuracy to call indels. This is critical if xAtlas is meant to be used in clinical settings.

We are happy to hear that the reviewer had no problems running xAtlas. We agree the realignment step is often important to obtain more accurate indel calling and also potentially in certain regions of the genome. Actually one can see this nicely as we upgraded our benchmarks to v4.2.1 from GIAB the performance in indels decreased because of the added repetitive regions. Nevertheless, the substitutions remain at a high level. We currently do not include a realignment step, but executed one for the bam file that is used by all the variant calling methods benchmarked here. Furthermore, we now extended the benchmark to the GIAB SNV v.4.2.1 which includes even more hard to assess regions of the gnome. Overall the performance of xAtlas remains fast and accurate, obviously with more CPU power spent this can be improved like in every other field of computational science.

2. The manuscript looks outdated in terms of evaluation datasets, metrics and available tools. Since high values of standard precision and sensitivity are easy to achieve with simple SNV callers, metrics such as the false positives per million basepair (FPPM) proposed by the developers of the synthetic diploid benchmark dataset should be used to achieve a more clear assessment of the accuracy of the different methods (https://urldefense.proofpoint.com/v2/url?u=https-3A__doi.org_10.1038_s41592-2D018-2D0054-2D7&d=DwIGaQ&c=ZQs-KZ8oxEw0p81sqgiaRA&r=hkDaFIlMMg5o5xD5aQ2Txzhw0BLHnUrK9Cxqzcl0pbM&m=EKbrYWb9s4S-H0LibUFSZHzVZUQl8HR8RNffFrgtXGQ&s=2E57xmF-RGwIwcYIDd00FOPbGonCizBpcMRjdKWRgtQ&e=). Regarding benchmark experiments, SynDyp should also be used for benchmarking. To actually support that xAtlas is reliable across heterogeneus datasets (as stated in the title), further datasets should be tested, as it has been done for software tools such as NGSEP (https://urldefense.proofpoint.com/v2/url?u=https-3A__doi.org_10.1093_bioinformatics_btz275&d=DwIGaQ&c=ZQs-KZ8oxEw0p81sqgiaRA&r=hkDaFIlMMg5o5xD5aQ2Txzhw0BLHnUrK9Cxqzcl0pbM&m=EKbrYWb9s4S-H0LibUFSZHzVZUQl8HR8RNffFrgtXGQ&s=akoXVF6idqbNKYRhHF7fw25evSk0lEJBAfJZylfr7Vs&e=). In terms of tools, both DeepVariant and NGSEP should be included in the comparisons.

We thank the reviewer for all these suggestions. We followed the best practices according to GIAB, a National Standards and Technology group. We have now extended our benchmark by including GIAB v. 4.2.1 benchmark that further includes hard-to-assess regions. xAtlas is meant to be a versatile and fast SNV caller, and as such DeepVariant and Dragen are slower (CPU times) or require specialized hardware or cloud infrastructure to obtain similar results. As an example, multiple research institutes don't allow docker to run on their clusters, and thus this can be a limitation where e.g. Dragen cannot be used.

3. Regarding the metrics proposed by the authors, I do not think it is a good practice to merge results on accuracy and efficiency, taking into account that the accuracy in this case is lower than other solutions, and for clinical settings that is an important issue. The supplementary table should also report sensitivity and precision for indels, not only for SNVs.

We politely disagree, we think it is interesting how good SNV calls one can obtain in a given time or with CPU constraints. We have adopted Figure 2 to make this clearer as now the fastest method is on the bottom. The speed and low requirements should enable small labs to analyze their own data and still maintain relatively high accuracy on their variant calls. It is true like in so many other fields that if you improve coverage, runtime, or specialized hardware that the results can be further improved and we clearly showcase this.

4. The SNV calling method and particularly the genotyping procedure should be describe in much better detail. The manuscript describes the general pileup process, then, it mentions some general filters for read alignments and then it mentions that it applies logistic regression. However, it is not clear which data is used for such regression or in general how allele counts and quality scores are taken into account. A much deeper description of the logistic regression model should be included in the manuscript.

We thank the reviewer for highlighting this and we improved the description by adding table 2 and extending the method section.

5. There are better methods than PCA to show clustering of the 1000g samples. A structure analysis is more suitable for population genomics data and it is more clear to show the different subpopulations.

This was just as a demonstration highlighting the overall correctness for xAtlas even on the population scale. We agree and a more detailed analyses over 1000g samples have been done and published previously.

6. Finally, about the software, genotype calls produced by the xAtlas should have a value for the genotype quality (GQ) format field to assess the genotyping accuracy. For single sample analysis the QUAL value can be used (although this is not entirely correct). However, for population VCFs, the GQ field is very important to have a measure of genotyping quality per datapoint. Regarding population VCF files it is not clear, either from the in-line help or from the github site, how population VCF files should be constructed.

While we understand the importance of GQ format field values for representing sample-level genotype accuracy in population-level variant analyses, GQ values are omitted because xAtlas does not calculate phred-scaled genotype quality scores as part of the variant evaluation process. QUAL values can be phred-scaled and used as a proxy for genotype quality if GQ values are still needed.

We have some additional comments from a 3rd reviewer who didn't submit a formal report. We would strongly recommend you address them as well to improve the paper.

Reviewer 3:

In this manuscript, the authors described xATLAS, a variant caller developed for SNV and indels.

Major comments

1) It's unclear to me what the advantage of this tool compared to other callers. The authors compared a variety of tools in Figure 2, but DeepVariant and DRAGEN are not on the list. These two variant callers are well-known for their fast performance.

We initially did not include Dragen or DeepVariant because they require additional resources that hindered them from being run locally. We have now extended the benchmark section by using GIAB v4.2.1 and also benchmarked DeepVariant. We could not get this to run on our local infrastructure as the installation is impossible and our cluster (as many others) don't allow us to run docker on it. Thus we downloaded the VCF file from Dragen over their submission to an FDA challenge.

DeepVariant precision and sensitivity relative to GIAB v4.2.1 benchmark small variants based on their precision FDA submission:

**Sample**	**Coverage**	**SNV precision**	**SNV sensitivity**	**Indel precision**	**Indel sensitivity**
HG002	~35	0.9982	0.9935	0.9736	0.9519
HG003	~35	0.9971	0.9971	0.9735	0.9521
HG004	~35	0.9972	0.9972	0.9730	0.9510

2) Third paragraph under findings: "The ideal variant caller would therefore manage both scalability issues and sources of data heterogeneity." What are the data heterogeneity issues

xATLAS can address? This isn't clear from the experiments / analyses performed.

We thank the reviewer for highlighting this and we reworded this now. “The ideal variant caller should therefore allow fast performance and scalability on commodity computing hardware”

3) What does "single-sample call sets" mean in the 3rd line of the third paragraph under findings?

We just wanted to indicate that xAtlas runs on a per bam file basis.

4) Page 5, the paragraph under description of Figure 1, a logistic regression model was built first to create a likelihood score, together with other filters to determine whether a variant is good or not. Why are the low alignment quality and other filter criteria not used together with other variables in the regression model?

Reads that xAtlas filters out prior to candidate evaluation, such as those with low alignment scores or those marked as duplicate reads, are generally not informative as evidence of sequence variation. We have now expanded on these details in the manuscript section.

5) Page 10, last paragraph: "Each VCF record in the pVCF file represents a genetic locus from the previously determined set of all xAtlas-called genetic loci across the sample set, and each of the 3202 samples represented by a sample-level column denoting the presence or absence of an xAtlas-called genotype at each genetic loci." Does that mean that the individual genotype 1/1, 0/1 and 0/0 were not reported any more? Absence or presence sounds like just a zero or one value.

We have clarified this description in the text. xAtlas called genotypes (0/0, 0/1, 1/1) are preserved in the final pVCF. Genetic loci without called genotypes for a given sample are denoted by “.”.

6) More information on how to run the xtlas is needed for the documentation page for the https://urldefense.proofpoint.com/v2/url?u=https-3A__github.com_jfarek_xatlas&d=DwIGaQ&c=ZQs-KZ8oxEw0p81sqgiaRA&r=hkDaFIlMMg5o5xD5aQ2Txzhw0BLHnUrK9Cxqzcl0pbM&m=EKbrYWb9s4S-H0LibUFSZHzVZUQl8HR8RNffFrgtXGQ&s=goGuqrkQSIx8DYVd4sPnOWcBYFaBQJgrlhDQ4plCMBg&e= is needed. There is no instruction on how to run the caller from CRAM to VCF or gVCF, also, there is no instruction on how the "project-level VCF (pVCF)" can be constructed from the individual level genotype data.

We thank the reviewer for highlighting this. We now extended the documentation and also added a toy example.

7) There are a few places where the users can tune their thresholds. Recommendations on how to use them, in what scenarios, will be extremely useful.

We thank the reviewer for this suggestion. We have improved our documentation on Github and the parameters are explained in the help message of the tool. It is hard to predict the circumstances that scientists face to deviate from our recommended parameters and thus we did not extensively discuss other parameter settings. As it is shown in the results and also over other user reports and publications (xAtlas being used in a production setting at Baylor) xAtlas default parameters are working very well across different data sets.

Minor comments

1) Under findings, third sentence starting with "Despite multiple xxx" seems incomplete. Same problem for the last sentence in the same paragraph.

Fixed.

2) Page 9, line 9, there is a huge "blank" after "SNVs"- is something missing there?

Fixed.
