# Supplementary material for: xAtlas: scalable small variant calling across heterogeneous next-generation sequencing experiments
Source: Gigascience. 2023 Jan 16;12:giac125. doi: 10.1093/gigascience/giac125 (PMC9841152; doi:10.1093/gigascience/giac125)
Supplement: giac125_Response_to_Reviewer_Comments_Original_Submission [file giac125_response_to_reviewer_comments_original_submission.pdf]

Author's Response To Reviewer Comments

Close

We submitted a detailed response to each of the reviewers comments in the cover letter.

Close
