# Supplementary material for: xAtlas: scalable small variant calling across heterogeneous next-generation sequencing experiments
Source: Gigascience. 2023 Jan 16;12:giac125. doi: 10.1093/gigascience/giac125 (PMC9841152; doi:10.1093/gigascience/giac125)
Supplement: giac125_Response_to_Reviewer_Comments_Revision_1 [file giac125_response_to_reviewer_comments_revision_1.pdf]

Author's Response To Reviewer Comments

Close

The details of the cover letter are in the attached document. We responded to each of the points rased and marked the responses in blue. We further tracked the changes in the main text in blue.
Thank you
Fritz Sedlazeck

Close
