# Supplementary material for: xAtlas: scalable small variant calling across heterogeneous next-generation sequencing experiments
Source: Gigascience. 2023 Jan 16;12:giac125. doi: 10.1093/gigascience/giac125 (PMC9841152; doi:10.1093/gigascience/giac125)
Supplement: giac125_Response_to_Reviewer_Comments_Revision_2 [file giac125_response_to_reviewer_comments_revision_2.pdf]

Author's Response To Reviewer Comments

Close

We thank the editor for clarifying our questions on the previous review replies. We have now updated Figure2 to show SNV, indel and combined (SNV&indel) performance for all methods. We hope that this addresses the last concerned that was mentioned.

Close
