# Supplementary material for: xAtlas: scalable small variant calling across heterogeneous next-generation sequencing experiments
Source: Gigascience. 2023 Jan 16;12:giac125. doi: 10.1093/gigascience/giac125 (PMC9841152; doi:10.1093/gigascience/giac125)
Supplement: giac125_Reviewer_1_Report_Original_Submission — Ruibang Luo -- 9/19/2021 Reviewed [file giac125_reviewer_1_report_original_submission.pdf]

Reviewer Report

Title: xAtlas: Scalable small variant calling across heterogeneous next-generation sequencing experiments

Version: Original Submission **Date:** 9/19/2021

Reviewer name: Ruibang Luo

Reviewer Comments to Author:

In this paper, the authors proposed xAtlas, an open-source NGS variant caller. xAtlas is a fast and lightweight caller with comparable performance with other benchmarked callers. The benchmark comparison in multiple popular short-read platforms (Illumina HiSeq X and NovaSeq) demonstrated xAtlas's capacity to identify small variants rapidly with desirable performance. Although xAtlas is limited to call multi-allelic variants, the high sensitivity (~99.75% recall for ~60x benchmarking datasets) and desirable runtime (<2 hours) enable xAtlas to rapidly filter candidates and be considered as important quality control for further utilization.The authors presented a detailed explanation of xAtlas's workflow, design decisions and have done complete experiments in benchmarking, while there are still some points the authors need to discuss further listed as follow:The authors reported the performance in multiple coverages of the HG001 sample and the benchmarking result of HG002-4 samples by measuring the concordance with the GIAB truth set (v3.3.2). I noticed that GIAB had updated the GIAB truth sets from v3.3.2 to v4.2.1 for the Ashkenazi trio. The updated version included more difficult regions like segmental duplications and the Major Histocompatibility Complex (MHC) to identify previously unknown clinically relevant variants. Therefore, it would be helpful if the author could give a performance evaluation using the updated truth sets to give a more comprehensive performance of the proposed caller.In the Methods section, The authors stated the main three stages of the xAtlas variant calling process: read prepossessing, candidates identification, and candidates evaluation. The author fed hand-craft features (base quality, coverages, reference and alternative allele support, etc.) into a logistic regression model to classify true variants and reference calls in the candidate evaluation stage. But in Figure 1, the main workflow of xAtlas, only model scoring was shown, and the evaluation details were not visible. It would be useful if the authors could enrich Figure 1 to add more details to ensure consistency with Methods and facilitate reader understanding.In Figure 2, the authors reported the xAtlas performance comparison across in HG001 dataset with other variant callers. I noticed that the x-axis was F1-score while the y-axis was true positives per second. The tendency measurement of two metrics seems irrelevant, which might confuse the readers. we suggest the authors make separate comparisons for the two metrics. (For instance, plot Precision-Recall curves for F1-score measurement and Runtime comparison of various variant callers for speed benchmarking).Zheng, Zhenxian on behalf of the primary reviewer

Level of Interest

Please indicate how interesting you found the manuscript: Choose an item.

Quality of Written English

Please indicate the quality of language in the manuscript: Choose an item.

Declaration of Competing Interests

Please complete a declaration of competing interests, considering the following questions:

- Have you in the past five years received reimbursements, fees, funding, or salary from an organisation that may in any way gain or lose financially from the publication of this manuscript, either now or in the future?
- Do you hold any stocks or shares in an organisation that may in any way gain or lose financially from the publication of this manuscript, either now or in the future?
- Do you hold or are you currently applying for any patents relating to the content of the manuscript?
- Have you received reimbursements, fees, funding, or salary from an organization that holds or has applied for patents relating to the content of the manuscript?
- Do you have any other financial competing interests?
- Do you have any non-financial competing interests in relation to this paper?

If you can answer no to all of the above, write 'I declare that I have no competing interests' below. If your reply is yes to any, please give details below.

I declare that I have no competing interests

I agree to the open peer review policy of the journal. I understand that my name will be included on my report to the authors and, if the manuscript is accepted for publication, my named report including any attachments I upload will be posted on the website along with the authors' responses. I agree for my report to be made available under an Open Access Creative Commons CC-BY license (http://creativecommons.org/licenses/by/4.0/). I understand that any comments which I do not wish to be included in my named report can be included as confidential comments to the editors, which will not be published.

Choose an item.

To further support our reviewers, we have joined with Publons, where you can gain additional credit to further highlight your hard work (see: https://publons.com/journal/530/gigascience). On publication of this paper, your review will be automatically added to Publons, you can then choose whether or not to claim your Publons credit. I understand this statement.

Yes Choose an item.
