# Supplementary material for: xAtlas: scalable small variant calling across heterogeneous next-generation sequencing experiments
Source: Gigascience. 2023 Jan 16;12:giac125. doi: 10.1093/gigascience/giac125 (PMC9841152; doi:10.1093/gigascience/giac125)
Supplement: giac125_Reviewer_2_Report_Original_Submission — Jorge Duitama -- 10/4/2021 Reviewed [file giac125_reviewer_2_report_original_submission.pdf]

Reviewer Report

Title: xAtlas: Scalable small variant calling across heterogeneous next-generation sequencing experiments

Version: Original Submission **Date:** 10/4/2021

Reviewer name: Jorge Duitama

Reviewer Comments to Author:

The manuscript describes a variant caller called xAtlas, which uses a logistic regression model to call SNPs after building an alignment and pileup of the reads. The manuscript is clear. The software is built with the aim of being faster than other solutions. However, I have some concerns relative to the method and the manuscript.1. Unfortunately, the biggest issue with this work is that the gain of speed is obtained with an important sacrifice in accuracy, specially to call indels. I ran xAtlas with two different benchmark datasets and the accuracy, especially for indels and other complex regions was about 20% lower compared to other solutions. Although the difference was smaller, xAtlas is also less accurate than other software tools for SNV calling. It is well known that even a simple SNV caller can achieve high sensitivity and specificity (see results from https://doi.org/10.1101/gr.107524.110). However, several SNV errors can be generated by incorrect alignment of reads around indels and other complex regions. For that reason most of the work on variant detection is focused on mechanisms to perform indel realignment or de-novo miniassembly to increase accuracy of both SNV and indel detection. The paper of Strelka is a great example of this (https://doi.org/10.1038/s41592-018-0051-x). The manuscript does not mention if any procedure has been implemented to realign reads or to increase in some way the accuracy to call indels. This is critical if xAtlas is meant to be used in clinical settings.2. The manuscript looks outdated in terms of evaluation datasets, metrics and available tools. Since high values of standard precision and sensitivity are easy to achieve with simple SNV callers, metrics such as the false positives per million basepair (FPPM) proposed by the developers of the synthetic diploid benchmark dataset should be used to achieve a more clear assessment of the accuracy of the different methods (https://doi.org/10.1038/s41592-018-0054-7). Regarding benchmark experiments, SynDyp should also be used for benchmarking. To actually support that xAtlas is reliable across heterogeneus datasets (as stated in the title), further datasets should be tested, as it has been done for software tools such as NGSEP (https://doi.org/10.1093/bioinformatics/btz275). In terms of tools, both DeepVariant and NGSEP should be included in the comparisons.3. Regarding the metrics proposed by the authors, I do not think it is a good practice to merge results on accuracy and efficiency, taking into account that the accuracy in this case is lower than other solutions, and for clinical settings that is an important issue. The supplementary table should also report sensitivity and precision for indels, not only for SNVs.4. The SNV calling method and particularly the genotyping procedure should be describe in much better detail. The manuscript describes the general pileup process, then, it mentions some general filters for read alignments and then it mentions that it applies logistic regression. However, it is not clear which data is used for such regression or in general how allele counts and quality scores are taken into account. A much deeper description of the logistic regression model should be included in the manuscript.5. There

are better methods than PCA to show clustering of the 1000g samples. A structure analysis is more suitable for population genomics data and it is more clear to show the different subpopulations.6. Finally, about the software, genotype calls produced by the xAtlas should have a value for the genotype quality (GQ) format field to assess the genotyping accuracy. For single sample analysis the QUAL value can be used (although this is not entirely correct). However, for population VCFs, the GQ field is very important to have a measure of genotyping quality per datapoint. Regarding population VCF files it is not clear, either from the in-line help or from the github site, how population VCF files should be constructed.

Level of Interest

Please indicate how interesting you found the manuscript: Choose an item.

Quality of Written English

Please indicate the quality of language in the manuscript: Choose an item.

Declaration of Competing Interests

Please complete a declaration of competing interests, considering the following questions:

- Have you in the past five years received reimbursements, fees, funding, or salary from an organisation that may in any way gain or lose financially from the publication of this manuscript, either now or in the future?
- Do you hold any stocks or shares in an organisation that may in any way gain or lose financially from the publication of this manuscript, either now or in the future?
- Do you hold or are you currently applying for any patents relating to the content of the manuscript?
- Have you received reimbursements, fees, funding, or salary from an organization that holds or has applied for patents relating to the content of the manuscript?
- Do you have any other financial competing interests?
- Do you have any non-financial competing interests in relation to this paper?

If you can answer no to all of the above, write 'I declare that I have no competing interests' below. If your reply is yes to any, please give details below.

I declare that I have no competing interests

I agree to the open peer review policy of the journal. I understand that my name will be included on my report to the authors and, if the manuscript is accepted for publication, my named report including any attachments I upload will be posted on the website along with the authors' responses. I agree for my report to be made available under an Open Access Creative Commons CC-BY license

(http://creativecommons.org/licenses/by/4.0/). I understand that any comments which I do not wish to be included in my named report can be included as confidential comments to the editors, which will not be published.

Choose an item.

To further support our reviewers, we have joined with Publons, where you can gain additional credit to further highlight your hard work (see: https://publons.com/journal/530/gigascience). On publication of this paper, your review will be automatically added to Publons, you can then choose whether or not to claim your Publons credit. I understand this statement.

Yes Choose an item.
