# Supplementary material for: xAtlas: scalable small variant calling across heterogeneous next-generation sequencing experiments
Source: Gigascience. 2023 Jan 16;12:giac125. doi: 10.1093/gigascience/giac125 (PMC9841152; doi:10.1093/gigascience/giac125)
Supplement: giac125_Reviewer_2_Report_Revision_1 — Jorge Duitama -- 3/7/2022 Reviewed [file giac125_reviewer_2_report_revision_1.pdf]

Reviewer Report

Title: xAtlas: Scalable small variant calling across heterogeneous next-generation sequencing experiments

Version: Revision 1 **Date:** 3/7/2022

Reviewer name: Jorge Duitama

Reviewer Comments to Author:

In this revision the authors updated the evaluation of their method to take into account the new version of the GAB benchmark and made adjustments in the text. Unfortunately, I have to say that I am not satisfied with the answers provided to my previous comments. Based on the answers to the comments of all reviewers, I have to say that, in its current status, I see very little applicability of xAtlas for variant detection, compared to other tools. In particular, I would not recommend xAtlas for analysis of clinical samples due to its lower accuracy to call all types of variants, compared to other solutions. Here is the status of each comment:1. Accuracy issues. The authors did not seem to make any improvement on the variant detection method to improve indel detection accuracy. The answer does not address this issue, but instead makes comments on the new version of the GIAB benchmark, which was not the point of the original concern. I agree that accuracy could be improved in future versions of the software but unfortunately the current accuracy is very far from the current state of the art.2. About evaluation datasets and benchmarked tools. This is the most critical point and my reasoning to recommend rejection. The authors limited their answer to update the results with the GAIB benchmark. Although I agree that the GAIB benchmark is a great effort to provide a dataset to improve variant calling in human samples, it is not the only effort. The authors completely ignored the suggestion of testing with the Syndip dataset or other and use their evaluation metrics such as FPPM. The related manuscripts were not even mentioned to at least provide a rationale on why these benchmark efforts were not considered. More important than that, the authors report indel accuracy but do not compare this accuracy against that of other tools. This comparison needs to be clearly reported to make clear the loss of accuracy that users need to take into account if they choose xAtlas for their analysis pipelines. Finally, about benchmarking tools, the authors did not report the accuracy of DeepVariant in the manuscript as the three reviewers requested. Moreover, they completely ignored my request to compare against NGSEP, even though NGSEP is also a tool with better efficiency than GATK but with a much lower penalty on accuracy (its accuracy is even superior in some particular cases)3. Reporting of efficiency and accuracy. The new figure 2 is more clear but (as mention above) the main issue is that indel accuracy should be reported and that at least NGSEP should be included.4. Method description. The authors improved the explanation of the method and provided a table with the parameters of the regression. The method is relatively simple (for that reason it is quick) and it may be over fitted for the GIAB dataset, but the comment was addressed5. Analysis of the 1000g data. The authors did not make any improvement here. I am not asking the reviewers to reproduce the complete results of population genomics achieved by the 1000g project. I understand, they want to show the 1000g data for benchmark purposes, but a PCA is a very crude way to achieve this. I encourage again the authors to at

least run a more informative method such as structure. Otherwise I think it is better to remove this section.6. Lack of GQ value. The authors did not make any improvement here. GQ values are very important in population genomics datasets to improve the accuracy of population genetic analysis. The GQ values reflect the confidence of each method to make each genotype call. GQ values are calculated and reported by all state of the art variant callers.

Level of Interest

Please indicate how interesting you found the manuscript: Choose an item.

Quality of Written English

Please indicate the quality of language in the manuscript: Choose an item.

Declaration of Competing Interests

Please complete a declaration of competing interests, considering the following questions:

- Have you in the past five years received reimbursements, fees, funding, or salary from an organisation that may in any way gain or lose financially from the publication of this manuscript, either now or in the future?
- Do you hold any stocks or shares in an organisation that may in any way gain or lose financially from the publication of this manuscript, either now or in the future?
- Do you hold or are you currently applying for any patents relating to the content of the manuscript?
- Have you received reimbursements, fees, funding, or salary from an organization that holds or has applied for patents relating to the content of the manuscript?
- Do you have any other financial competing interests?
- Do you have any non-financial competing interests in relation to this paper?

If you can answer no to all of the above, write 'I declare that I have no competing interests' below. If your reply is yes to any, please give details below.

I declare that I have no competing interests

I agree to the open peer review policy of the journal. I understand that my name will be included on my report to the authors and, if the manuscript is accepted for publication, my named report including any attachments I upload will be posted on the website along with the authors' responses. I agree for my report to be made available under an Open Access Creative Commons CC-BY license (http://creativecommons.org/licenses/by/4.0/). I understand that any comments which I do not wish to be included in my named report can be included as confidential comments to the editors, which will not be published.

Choose an item.

To further support our reviewers, we have joined with Publons, where you can gain additional credit to further highlight your hard work (see: https://publons.com/journal/530/gigascience). On publication of this paper, your review will be automatically added to Publons, you can then choose whether or not to claim your Publons credit. I understand this statement.

Yes Choose an item.
