# Supplementary material for: xAtlas: scalable small variant calling across heterogeneous next-generation sequencing experiments
Source: Gigascience. 2023 Jan 16;12:giac125. doi: 10.1093/gigascience/giac125 (PMC9841152; doi:10.1093/gigascience/giac125)
Supplement: giac125_Reviewer_4_Report_Revision_1 — Lachlan Coin -- 8/28/2022 Reviewed [file giac125_reviewer_4_report_revision_1.pdf]

Reviewer Report

Title: xAtlas: Scalable small variant calling across heterogeneous next-generation sequencing experiments

Version: Revision 2 **Date:** 8/28/2022

Reviewer name: Lachlan Coin

Reviewer Comments to Author:

Previously I gave some comments as an EBM which I am including here for completeness:I note the correspondence from Justin Zook. I agree that the latest GIAB benchmark is sufficient as it incorporates long and linked reads, and it may not be necessary to use SynDyp in addition.However, the second reviewer makes some strong points in addition to this relatively minor suggestion to use SynDyp, which I feel the authors should address before the manuscript is re-consideredThe reviewer points (and the authors acknowledge) the performance of xAtlas is not good on indels. However, the main figure (figure 1) obscures this by plotting a combined concordance F measure (across SNPs + indels) vs runtime.I find it highly unusual to combine runtime and accuracy in a single plot. This was pointed out by the reviewer. I do see the authors point that you might wish to sacrifice accuracy for runtime, but plotting them in the same figure is confusing, and non-standard.It is more standard to plot precision vs sensitivity in one plot (with different symbols for SNPs and Indels respectively); and to show runtime in a separate panel. This provides more information needed to users to decide which tool best suits them.The reviewer points to this figure from NGSEP3 as a good way to show all the information on runtime and accuracy https://academic.oup.com/view-large/figure/175268621/btz275f3.tif (in fact in this figure more information is shown by varying the minimum GQ score, I realise this is not possible here).I would not ask the authors to use FPPM , its fine to use precision (FPPM is good for comparing across species, which is not relevant here).The reviewer asked to include DeepVariant and NGSEP in the comparisons. I think this is reasonable. The authors have obtained information on sensitivity and precision for DeepVariant, but I guess not runtime. The DeepVariant results are currently included only in Supplementary and I dont think are even referenced in the manuscript. The authors could show DeepVariant SNV and indel precision and sensitivity in the sensivitiy vs precision plot suggested in point 1, and note that they do not have runtimes .With regard to NGSEP, I find it reasonable to request this be included in the comparison, as the paper (https://doi.org/10.1093/bioinformatics/btz275) describing it shows that it has better accuracy than Strelka and is also substantially faster. So it is addressing a similar space to xAtlas. Also it has the advantage of producing GQ scores.With regard to GQ scores, it is unfortunate xAtlas does not provide these, and this needs to be clearly addressed in a limitations section that they are not able to be calculated.With regard to the 1000 genomes PCA, I agree with the reviewer that this analysis is not very informative. At least they should show a comparison to the PCA obtained from a more accurate method? There are many more informative analyses here e.g. genotype correlation between xAtlas and gold-standard population level genotype calls would be far more informative.Now, I am providing comments after the authors responded to the above points. I thank the authors for addressing many of these points.The one remaining issue is that in Figure 2b they still show the precision/recall over all

variants, and I think they should show it also on SNVs and indels separately. Also in the abstract they just mention the precision recall on SNVs and not on indels. Again they should say the results of both in the abstract.

Level of Interest

Please indicate how interesting you found the manuscript: Choose an item.

Quality of Written English

Please indicate the quality of language in the manuscript: Choose an item.

Declaration of Competing Interests

Please complete a declaration of competing interests, considering the following questions:

- Have you in the past five years received reimbursements, fees, funding, or salary from an organisation that may in any way gain or lose financially from the publication of this manuscript, either now or in the future?
- Do you hold any stocks or shares in an organisation that may in any way gain or lose financially from the publication of this manuscript, either now or in the future?
- Do you hold or are you currently applying for any patents relating to the content of the manuscript?
- Have you received reimbursements, fees, funding, or salary from an organization that holds or has applied for patents relating to the content of the manuscript?
- Do you have any other financial competing interests?
- Do you have any non-financial competing interests in relation to this paper?

If you can answer no to all of the above, write 'I declare that I have no competing interests' below. If your reply is yes to any, please give details below.

I declare that I have no competing interests

I agree to the open peer review policy of the journal. I understand that my name will be included on my report to the authors and, if the manuscript is accepted for publication, my named report including any attachments I upload will be posted on the website along with the authors' responses. I agree for my report to be made available under an Open Access Creative Commons CC-BY license (http://creativecommons.org/licenses/by/4.0/). I understand that any comments which I do not wish to be included in my named report can be included as confidential comments to the editors, which will not be published.

Choose an item.

To further support our reviewers, we have joined with Publons, where you can gain additional credit to further highlight your hard work (see: https://publons.com/journal/530/gigascience). On publication of this paper, your review will be automatically added to Publons, you can then choose whether or not to claim your Publons credit. I understand this statement.

Yes Choose an item.
